# Supplementary material for: Flavodiiron-mediated O2 photoreduction at photosystem I acceptor-side provides photoprotection to conifer thylakoids in early spring
Source: Nat Commun. 2023 Jun 3;14:3210. doi: 10.1038/s41467-023-38938-z (PMC10239515; doi:10.1038/s41467-023-38938-z)
Supplement: Supplementary file 1 — Supplementary Information [file 41467_2023_38938_MOESM1_ESM.pdf]

Supplementary information for,

Flavodiiron-mediated O_2 photoreduction at photosystem I acceptor-side provides photoprotection to conifer thylakoids in early spring

Authors: Pushan Bag[1, 6, &], Tatyana Shutova[1, &], Dmitry Shevela[2], Jenna Lihavainen[1], Sanchali Nanda[1], Alexander G. Ivanov[3, 4], Johannes Messinger[2, 5], Stefan Jansson[1, *]

Affiliations: [1]Umeå Plant Science Centre, Department of Plant Physiology, Umeå University, Umeå, Sweden, [2]Department of Chemistry, Chemical Biological Centre, Umeå University, Umeå, Sweden, [3]Department of Biology, University of Western Ontario, London, Ontario, Canada, [4]Institute of Biophysics and Biomedical Engineering, Bulgarian Academy of Sciences, Sofia, Bulgaria, [5]Department of Chemistry – Ångström laboratory, Uppsala University, Uppsala, Sweden
[6]Current address: Section of Molecular Plant Biology, Department of Biology, University of Oxford, Oxford, United Kingdom

[&]These authors contributed equally to this work

*Corresponding author (stefan.jansson@umu.se)

This supplementary file includes,

Supplementary tables 1-5
Supplementary figures 1-11
Supplementary references 1-6

Supplementary tables:

Supplementary table 1. All sampling and experiments that has been performed on either intact needles or isolated thylakoid membranes from pine and spruce over three seasons. On each date needles were harvested for either intact needle measurements or for isolation of thylakoid from 3-5 individual spruce and pine trees. Exact n for each figure/table is provided with the legend. Color on dates denotes seasons, i.e., early spring (blue) and summer (green). The season specific differences between early spring and summer samples/dates were consistent in all experiments within all three years, regardless of number of replicates/experiments. 'Yes' cells indicate which experiments were performed with a particular sample and blank cell means a particular experiment was not performed with a particular sample. A particular experiments not performed with a particular sample was associated with sample availability (ran out of samples) and did not mean failed experiment. All experiments were always performed, and data represented in the manuscript, from at least three (if more replicates were used in specific experiments those are mentioned in the legends) independent biological replicates, unless biological replicates pulled together. In such case samples were pulled from three-five biological replicates and two-three independent experiments (not technical replication of the same sample) were performed with the pulled samples. The table shows the total number of samples and experiments and repetitions to reach the conclusion. However, for maintaining homogeneity among all the represented data, the 2018 ES and S samples that were subjected to most (if not all) of the experiments are only considered for representation of the study and are marked with yellow fill.

Date	Sample ID	Sampled speceies	Clarke	MIMS	P700	PQ pool	Fluorescence	Immunoblot
2017-02-24	Samples identified with respective dates and not IDs	Pine,	Yes	-	Yes	-	Yes	-
2017-03-06		Pine, Spruce	Yes	Yes		-	Yes	-
2017-03-09		Pine, Spruce	Yes	Yes	Yes	-	Yes	-
2017-03-17		Pine	Yes	-	Yes	-	Yes	-
2017-06-03		Pine	Yes	Yes	Yes	-	Yes	-
2017-07-27		Pine, Spruce	Yes	Yes	Yes	-	Yes	-
**2018-02-12**	**ES1**	Pine, Spruce	Yes	Yes	Yes	Yes	Yes	Yes
**2018-02-25**	**ES2**	Pine, Spruce	Yes	Yes	Yes	Yes	Yes	Yes
**2018-03-12**	**ES3**	Pine, Spruce	Yes	Yes	Yes	Yes	Yes	Yes
**2018-07-15**	**S1**	Pine, Spruce	Yes	Yes	Yes	Yes	Yes	Yes
**2018-07-24**	**S2**	Pine, Spruce	Yes	Yes	Yes	Yes	Yes	Yes
2020-02-26	ES4	Pine, Spruce	Yes	Yes	Yes	-	-	Yes
2020-03-09	ES5	Pine, Spruce	Yes	Yes	-	-	-	Yes
2020-06-21	S3	Pine	Yes	-	Yes	-	-	Yes
2020-07-15	S4	Pine, Spruce	Yes	Yes		-	-	Yes

Supplementary table 2. PQ pool metabolite detection and analysis of thylakoid prenylquinones. Prenylquinones were analysed from thylakoids isolated from pine needles using UPLC-APCI(-)QTOF-MS/MS. The arrows mark the direction of the change in the metabolite level after a treatment with ascorbate (reducing agent). The redox state of prenylquinones were expressed as the ratio of reduced and oxidized forms calculated from the areas of the respective peaks. PQH2/PQ is shown in Fig. 2e. The statistical test results for the effect of season on the plastoquinone and ubiquinone levels and ratios are shown in Table 5.

	Metabolite	Abbreviation	Formula	Mass	RT (min)	APCI(-)		Annotation level	Ascorbate treatment
						Ion	m/z		
**1**	alpha-Tocopherol-quinone	α-TQ	C29H50O3	446.3760	2.18	[M]-	446.3760	MS/MS	↓
**2**	alpha-Tocopherol	α-T	C29H50O2	430.3811	2.49	[M-H]-	429.3733	standard	↑
**3**	Trimethylphytylbenzoquinone	TMPBQ	C29H48O2	428.3654	2.62	[M]-	428.3654	MS/MS	↓
**4**	Phylloquinone	K	C31H46O2	450.3498	3.04	[M]-	450.3498	MS/MS	-
**6**	Hydroxyplastochromanol	PC-OH	C53H81O3	765.6186	3.56	[M-H]-	764.6108	MS/MS	↑
**5**	Plastoquinol	PQ-9H2	C53H82O2	750.6315	3.72	[M-2H]-	748.6158	MS/MS	↑
**7**	Hydroxyplastoquinone	PQ-OH	C53H80O3	764.6108	3.75	[M]-	764.6108	MS/MS	↓
**9**	Plastochromanol-8	PC-8	C53H82O2	750.6315	5.01	[M-H]-	749.6237	MS/MS	↑
**8**	Ubiquinol	UBQH2	C59H92O4	864.6996	5.15	[M-2H]-	862.6839	MS/MS	↑
**10**	Plastoquinone-9	PQ-9	C53H80O2	748.6158	6.24	[M]-	748.6158	MS/MS	↓
**11**	Ubiquinone	UBQ	C59H90O4	862.6839	6.72	[M]-	862.6839	MS/MS	↓

Supplementary table 3. Identity of flavodiiron proteins in spruce. Gene ID, peptide sequence and eukaryotic orthologs (*Physcomitrella patens* and *Chlamydomonas reinhardtii*) of spruce FLVA, FLVB proteins (As shown in the eukaryotic cluster in the unrooted phylogenetic tree of flavodiiron proteins in *Supplementary Fig 8B*). (See *Supplementary dataset 1* for list of the Flv protein sequences in other organisms).

Gene ID	Peptide sequence (N-terminal manually cut)	Orthologs
MA_9941245g0010<br>[Picea abies]	**>comp74265_c0_seq1 FLVA, FLAVODIIORN PROTEIN A**<br>MANATAYICGSSPLNRSASALSGGQVSPYSLFRPGYLANGVFGANSYNDREFKNVCFGKKRLEKQCLSSFKFTIQASDTTSGALISSPTDQS<br>PPATEKRDSHVRIVNITNDTLGLRACSYERLKFEVEYGLKKGTTDNSYIIKGPTEMALIDVPDQAFTKEYIRILMSTEDVHLLKYLIIGHLSPKRIE<br>SVIALGESLAQKSLPLDVYCSNPAAQLLSVSIPENLKDVFSLKIVRAGDSLDLGGGHKLQFILIPTPRWPDGMCTYDPATQLIFTQKFFSAHVC<br>SDNDFDIGGWELYGEDWHFFYDCMLSPAAQQADAALKKLPIVAQFAKPSYTGKMGIDIVKTDVKYILSTMLSSLNLPVHISSTVSGPLRTEGV<br>FVTAAICPIHGPIVRYTLTELVREYNDWTNQRLRRTDEATIAVIYASAYGNTAALAQAISRGISKAGIGVETLNCELSTDEEVVSLVRRCNGFVIG<br>SPTLAGHMPTPIQKALGVILNDNEARPKPCGVFGSFGWSGEAVDEIEQRLKDAGFSFSFPTVRCKFKPTEGMLQICEESGTDIAQAVRKSKL<br>RQRRDASQFVMASNVDQAVGRVIGSLCVVSAMNGDAESAMVASWVSQASFVPPGITLAVAKDRAVESLILPGSKFILNVLGDGKSSAIMKQL<br>LKPFKPGEPRFEGLKTKQASSGGRIVLDAISWMECTVKSRMESGDHWVLYATVEDGQLQDDKTLTALHYRKTGTRY	Pp3c14_14450V3.2,<br>Cre12.g531900
MA_10436657g0010<br>[Picea abies]	**>comp49004_c0_seq2 FLVB, FLAVODIIORN PROTEIN B**<br>MAVSTSALGTYSWMRTSSSPMAFELFTRKPKLQSFDRVHRGLFWPTLKKPTVTRSGRKPKPSISMSLSNVNVLEKRRLQTQTITSIAPDVTTI<br>RSLDWDRDRFDIEFGLQNGTTYNSFIINADKLTIIDASHEKFKGLYLSAIKEHIDPSAIEYIIANHTEPDHSGLIPDLLDLAPNATVVGSKTCIIFLQ<br>NLVLRPFKSLVVKGGSTLDLGRGHLLEFVMAPNLHWPDTMFTLDHGAGILFTCDAFGMHYCSELLYDEDLGAIEPHYRFYYDCLMRPNCRS<br>VITALKRIAEKDFSIIATGHGPLLRYNVAELLNKYETWSKQALEKQLATVAVLYVSDYGFSDRLSQTLARGLTKTNTGVEMMDLNVVDIQDMIE<br>CVSRCAGVVIMAPPSSGPANKAITTIYAAVKPKQPVVIAESYGGDDEPVDTIVQRFVALGISLALPPLRIKEIPNEATYQQFEEAGTDLGQLLSQ<br>KKSIEAKKAGMPINVAKAIARISGGLYVVTAAKGSSNGAMVASWVSQASFKPLGITIAVAKDRAIESLMQVGDTFVLNCLEDGNYAPLMKHFLK<br>RFPPGADRFEGVQWVPAASNGSPILIDCIAYLECKVVKRMETNDHWIAYSHVSDGNVSKPQARTASHHRKIGNYY	Pp3c1_26720V3.2,<br>Cre16.g691800

Supplementary table 4. Relative abundance of FlvA protein in ES and S samples in pine and spruce. Here summer sample S1 was loaded in 25%, 50% and 100% for quality control, and all other samples loaded on 100% basis, where 100% corresponds to 4 µg of chlorophyll content in each sample. For quantification, S1 100% band intensity in each blot is considered as 1 relative unit (r.u) and rest of the band intensities were normalized based on the 1 r.u. of S1. Hence, S1 does not have any SD. Avg ± SD is obtained from 4 (For pine) and 3 (For spruce) independent immunoblots. Representative immunoblots of pine and spruce thylakoid are shown in Fig 3D and in Supplementary Fig 8A. The effect of sampling day was tested with one-way ANOVA and season with t-test. FlvA abundance was significantly affected by sampling day in pine (F 13.68, P=6.82e-05) and in spruce (F 13.14 P=5.42e-04) and by season (pine: t= -9.031, df=13.9, P=3.43e-07, spruce: t=-5.31, df=13, P=1.41e-04). Pairwise comparisons were performed with Fisher’s Least Significant Difference test (LSD), see the details of statistical results in Supplementary dataset 2. Different letters indicate significant difference between means (P-value <0.05). Source data are provided as a Source Data file.

Season	Sample ID	Chlorophyll in each lane	Relative abundance	SD	ANNOVA
**Pine**					
**Summer**	**s1**	25%	0.27	± 0.15	-
	**s1**	50%	0.53	± 0.12	-
	**s1**	100%	1.00	-	A
	**s2**	100%	1.27	± 0.17	A
**Early spring**	**e1**	100%	2.89	± 0.53	B
	**e2**	100%	3.08	± 0.43	B
	**e3**	100%	2.69	± 0.74	B
**Spruce**					
**Summer**	**s1**	25%	0.37	± 0.17	-
	**s1**	50%	0.48	± 0.03	-
	**s1**	100%	1.00	-	A
	**s2**	100%	1.04	± 0.10	A
**Early spring**	**e1**	100%	1.81	± 0.19	B
	**e2**	100%	1.43	± 0.14	C
	**e3**	100%	1.49	± 0.15	C

Supplementary table 5. The effect of season (summer S vs. early spring ES, n=9), on the relative abundance of prenylquinones and their ratios in pine needles was tested with two t-test (IBM SPSS Statistics). P-value <0.05 was considered significant. where n defines independent measurement replication(n=3) of individual biological replicates(n=3). Source data are provided as a Source Data file.

	**t**	**df**	**P-value**
**PQ**	7.99	10.41	9.00E-05
**PQH2**	-7.99	10.41	9.00E-05
**PQ/PQH2**	3.97	8.07	4.06E-03
**UBQ**	5.97	16.00	2.00E-05
**UBQH2**	-5.97	16.00	2.00E-05
**UBQ/UBQH2**	3.81	8.77	4.36E-03

Supplementary figures:

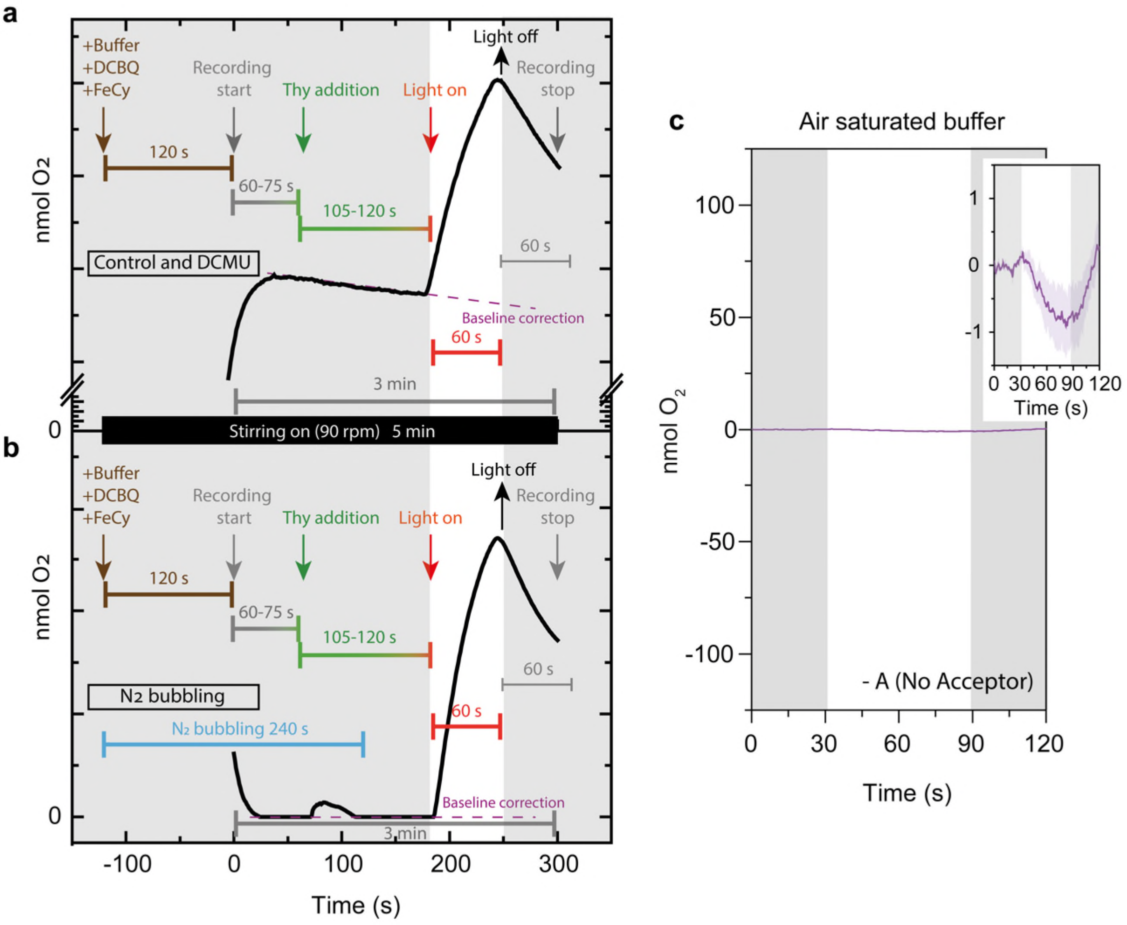

Supplementary Figure 1: Experimental design for Clark-type electrode O_2 measurements on isolated spinach thylakoid membranes suspended in air (a) and N_2 bubbled (b) buffer in presence (a, b) and absence (c) of exogenous electron acceptors, PPBQ and FeCy. Each individual experiment was carried out for 6 min and 40 s. First 250 µl of thylakoid suspension buffer was added in the Clarke electrode chamber, followed by addition of 30 µM PPBQ and 50 µM FeCy. Magnetic stirrer was switched on at a speed of 90 rpm. After 120 s instrument recording was started. Thylakoid membranes corresponding to 50 µg of chlorophyll were added after 60 s. After 105-120 s illumination with 800 µmol of photons m^{-2} s^{-1} white LED was switched on. With illumination, an increase/decrease in the yield of O_2 could be observed. After 60 s of illumination, the LED was switched off and recording was continued for 60 s before it was stopped. In case of N_2 bubbling, after adding PPBQ and FeCy, the chamber was closed and a continuous flow of N_2 was provided in the Clarke electrode chamber. Thylakoid samples were added after the O_2 yield reached zero (no O_2 in the chamber). Addition of thylakoid increased the O_2 yield slightly, that eventually reached again to zero within few seconds and then N_2 bubbling was stopped. Then the illumination was started for recording the light dependent O_2 yields from the thylakoid membranes. Baseline was calculated from the linear range of O_2 yields prior to illumination but after thylakoid addition to the Clarke electrode chamber. The colored shaded region following the O_2 yield curve (mean) indicates ±SEM (n=3) where n=number of biological replicates. Inset in **(c)** shows very minor O_2 activity with 100-fold zoom in y-axis compared to regular axis in **(c)**. Source data are provided as a Source Data file.

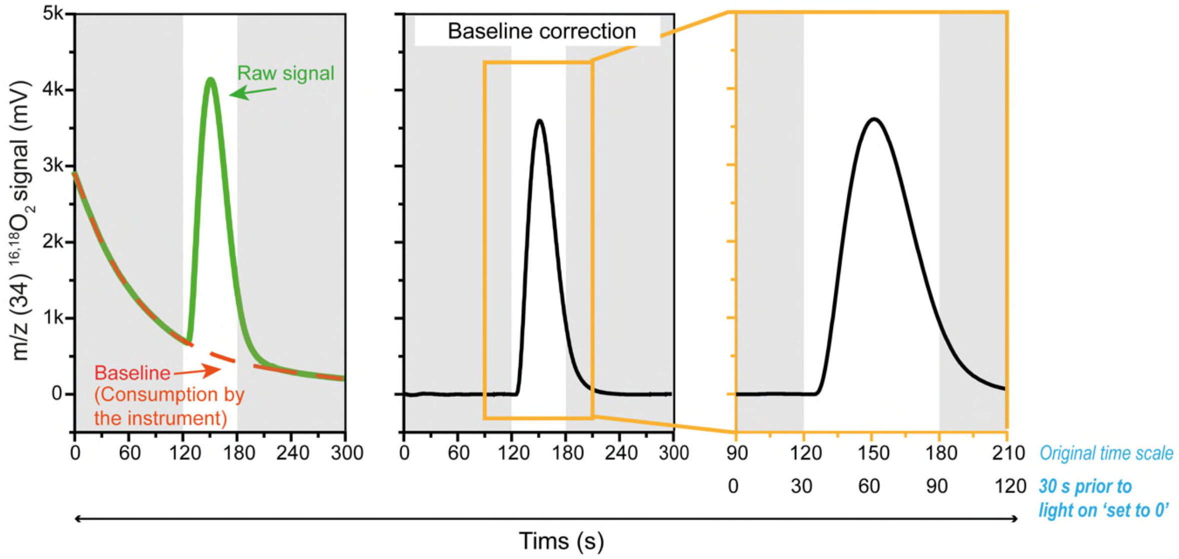

Supplementary Figure 2: Experimental details of MIMS assays. (a) Example of TR-MIMS signal processing. The raw $^{16,18}O_2$ signal was recorded for ~ 5 min by using TR-MIMS, where illumination was provided during the ~3rd min (from 120s-180s). The baseline {represents the $^{16,18}O_2$ consumption only by the instrument, Baseline was calculated using a 2nd polynomial order Savitzky-Golay filter [1] from whole the dark period before illumination and from the last 10s of the whole recording, with a filter window of 3 and threshold was set to 0.05 (In Savitzky-Golay algorithm, each data point is computed from data points within a moving window. If $\{\boldsymbol{f_i} | \boldsymbol{i} = \mathbf{1}, \mathbf{2}, \ldots, \boldsymbol{N}\}$ be the input data points and if $\{\boldsymbol{g_i} | \boldsymbol{i} = \mathbf{1}, \mathbf{2}, \ldots, \boldsymbol{N}\}$ denote the output data points. Each ***gi*** is computed from,

$$\{\boldsymbol{f_m} | \boldsymbol{i} - \boldsymbol{floor}(\boldsymbol{npts}/\mathbf{2}) < \boldsymbol{m} < \boldsymbol{i} + \boldsymbol{floor}(\boldsymbol{npts}/\mathbf{2})\}. \qquad \textbf{.... eq 1.1}$$

Where ***npts*** is the value of the Points of Window variable. The Savitzky-Golay method performs a polynomial regression to the data points in the moving window. Then ***gi*** will be computed as the value of the polynomial at position ***i***) (Left graph). Baseline extrapolates as a b-spline curves as in eq 1.2. Given ***n + 1*** control points ***P_0, P_1, ..., P_n*** and a knot vector ***U = { u_0, u_1, ..., u_m }***, the B-spline curve of degree ***p*** defined by these control points and knot vector ***U*** is

$$\boldsymbol{C}(\boldsymbol{u}) = \sum_{i=0}^{n} \boldsymbol{N_{i,p}}(\boldsymbol{u})\boldsymbol{P_i} \qquad \textbf{.... eq 1.2}$$

The baseline was then subtracted from the Raw signal (middle graph) and only 90th s to 210th s was shown in the main and supplementary figures. The scale was set to 0 at the 30s prior to illumination (right graph). The whole baseline correction analysis of the data was performed using Origin Pro 2021 standard baseline processing tool.

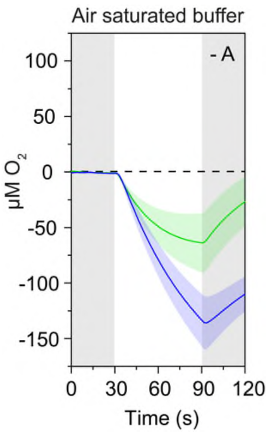

Supplementary Figure 3: O_2 yields in summer (S) and early spring (ES) pine thylakoid membranes in air saturated buffer in absence of exogenous electron acceptors, 250 µM PPBQ and 500 µM FeCy (-A). The colored shaded region following the O_2 yield curve (mean) indicates ±SEM (n=3) where n=number of biological replicates. In all gas exchange experiments data represent O_2 exchange corresponding to 50 µg of chlorophyll (For experimental design see Supplementary. Fig. S1A). Source data are provided as a Source Data file.

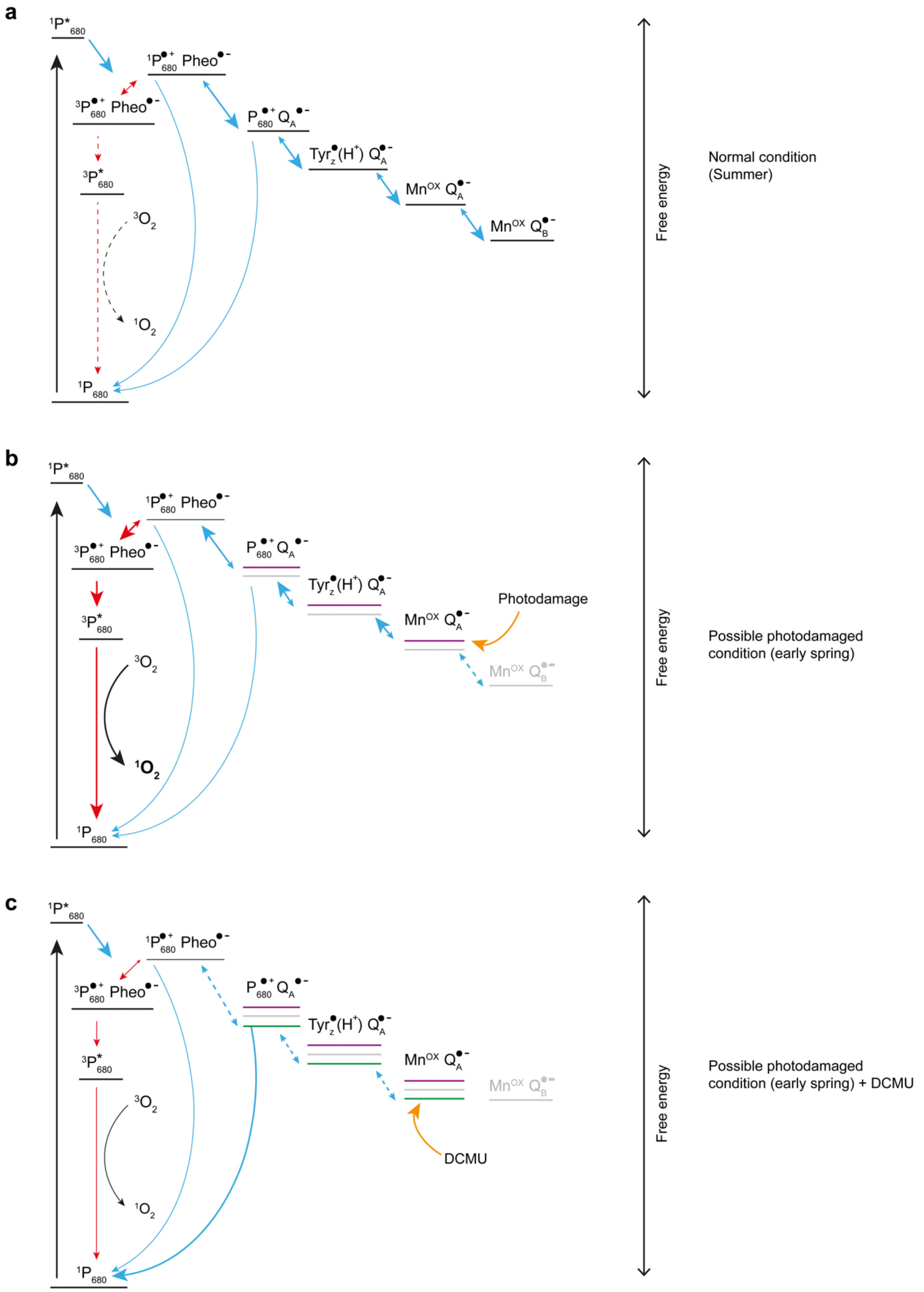

Supplementary Figure 4: Schematics of electron flow within PSII. (a) schematics represent the electron transfer reactions in PSII triggered upon illumination. A series of radical pairs are formed though which electrons are successively transferred forward and ultimately reach Q_B site and subsequently reduce a PQ bound to Q_B and is released as PQH_2 in the lumen. Each radical pair has

slightly lower energy (on the free energy scale) from the previous one (energy levels are denoted with black lines under each radical pair). Therefore, favors the forward electron transfer. This stabilizes the charge separation within PSII, such as, after excitation of $^1P_{680}$ to $^1P^*_{680}$, in step 2, $^1P_{680}^{\bullet+}$ $Pheo^{\bullet-}$ transfers an electron to $P_{680}^{\bullet+}$ $Q_A^{\bullet-}$ and then then the electron is transferred to the next pair and so on. If transfer of electron from $^1P_{680}^{\bullet+}$ $Pheo^{\bullet-}$ to next radical pair is limited, then charge recombination may take place and form $^3P_{680}^{\bullet+}$ $Pheo^{\bullet-}$ (Triplet) via spin conversion. This $^3P_{680}^*$ can convert oxygen to $^1O_2$ and thereby come back to the ground state, i.e., $^1P_{680}$. This triplet formation may also occur due to charge recombination with other electron acceptors, if in any step, the energy gap between any two of the radical pairs decreases then the radical pair may not be able to transfer the electron to the next pair and may transfer electron to the previous radical pair. Therefore, favoring a back reaction and triplet formation and conversion of oxygen to $^1O_2$, . $^1O_2$ reacts to proteins leading to damage and this phenomenon leads to O_2 consumption, which can be measured by monitoring yield of O_2.

(b) It has been shown that photodamage of the reaction centre may occur at the donor side (Mn cluster) or at the acceptor-side (Q_A). Under strong white light photodamage majorly occurs at Q_A, whereas UV light induces Mn cluster damage. Therefore, it can be assumed that natural light induced photodamage mainly occur at Q_A, as natural sunlight at the earth surface contains much lesser UV. In white light, the redox potential of Q_A decreases (Magenta lines) and therefore, in one hand, Q_A cannot transfer electron to Q_B anymore and on other hand the energy gap between radical pairs become smaller, which in turn favors back reaction and formation of long lived $^3P_{680}^*$. This will favor the chances $^1O_2$ formation by spin conversion of $^3O_2$ [2]–[4].

(c) The redox potential of Q_A can be modulated by addition of 3-(3′, 4′-dichlorophenyl)-1, 1-dimethylurea (DCMU), which binds to Q_A and increases the redox potential (Green line). This means the energy gap between $^1P_{680}^{\bullet+}$ $Pheo^{\bullet-}$ to will increase in presence of DCMU. Therefore, the probability of the back reaction form $P_{680}^{\bullet+}$ $Q_A^{\bullet-}$ to $^1P_{680}^{\bullet+}$ $Pheo^{\bullet-}$ will be very low. Rather the chances of non-radiative charge recombination to the ground states will be much higher. Therefore, production of $^1O_2$ from oxygen will be lower as well. Similar situation also occurs if the PSII reaction centre is Mn depleted [5].

Therefore, if indeed the $^{16}O_2$ consumption in ES samples was a manifestation of damaged reaction centre (Either at Mn cluster or at Q_A) then, addition of DCMU would decrease the redox potential of Q_A and therefore we should see decrease in $^{16}O_2$ consumption.

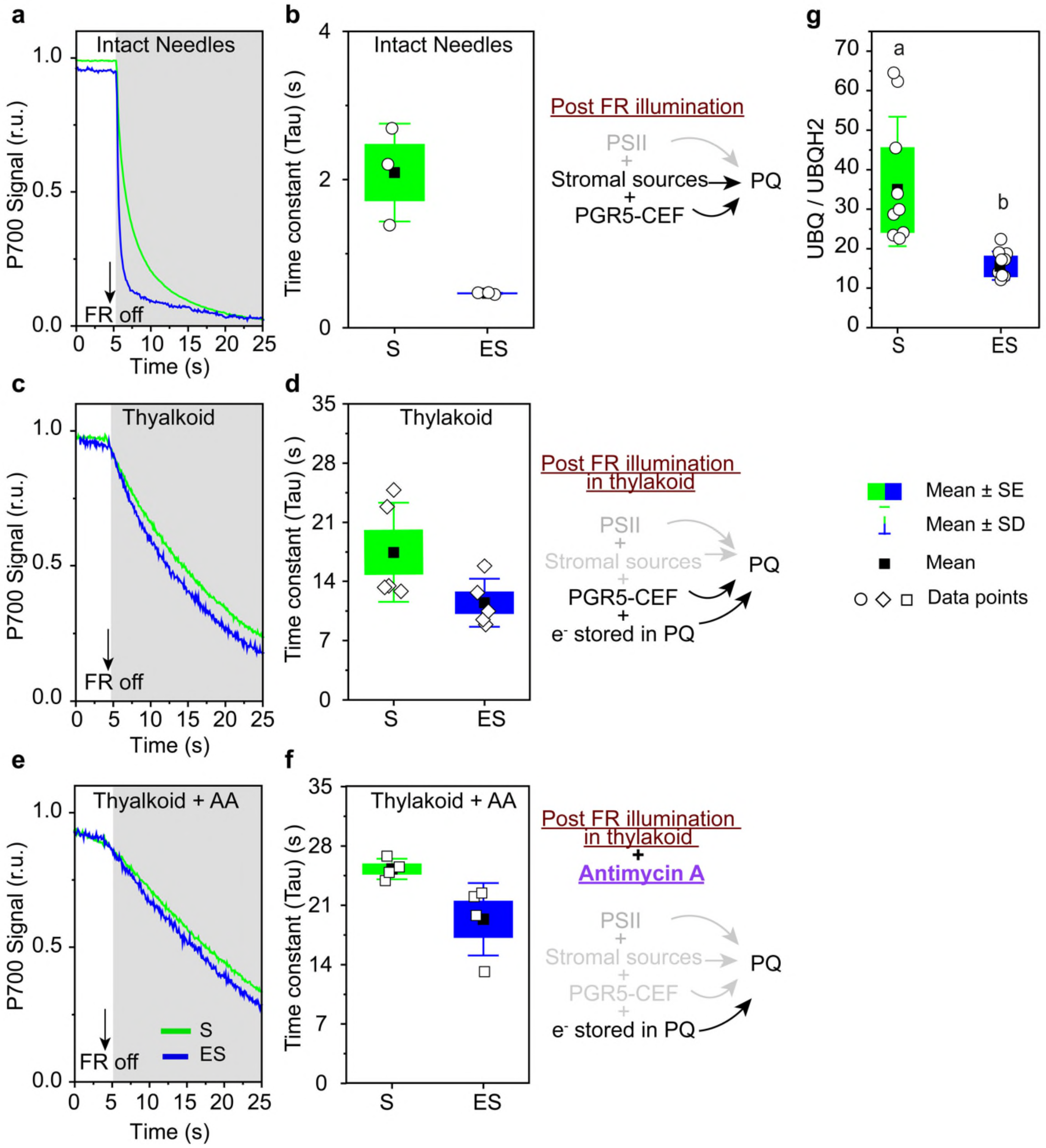

Supplementary Figure 5: P700 re-reduction and redox state of the ubiquinone pool. Re-reduction kinetics **(a, c, e)** of P700 signal followed by FR-light illumination and time constant (Tau) of the re-reduction **(b, d, f)** measured in vivo in intact pine needles **(a, b)** (n=3, n=number of measurements where needles were pulled from 3-5 trees), thylakoid membranes **(c, d)** (n=5) and 30 µM Antimycin A supplemented thylakoid membranes **(e, f)** (n=4), where n=number of biological replicates. In **b, d** and **f** the box bounds indicate ±SEM, minima/maxima indicate ±SD, black dot in the box indicates mean and white dots indicate data points. Since, Tau is a calculated parameter and not directly measured, therefore, no statistics have been derived. Schematics of possible electron donation pathways to PQ are shown adjacent to the graphs with respect to condition/treatment. P700 re-reduction phase post FR illumination, is marked by dark shaded regions. **(g)** Ratio of UQ and UQH2 in pine thylakoid membranes measured using UPLC-APCI(-)QTOF-MS (n=9) (see Supplementary. Table S3) where n defines independent measurement replication(n=3) of individual biological replicates(n=3). Two tailed t-test was performed for statistically significant mean differences (see Supplementary Table 5 for statistical details).

For the time constant Tau (s) calculation, the P700 traces were fitted with one-phase exponential function as reported previously [6] by using Origin Pro 2023.

The function used for fitting intact needles measurements,

$$y = \begin{cases} Yb & x < TD \\ Yb + A\,(1 - e^{-\left(\frac{x-TD}{Tau}\right)} & x \geq TD \end{cases} \qquad \text{... eq 3.1}$$

where TD = Time offset : x value at which exponential begins; Yb = Baseline : y value at which exponential begins, A = Amplitude : change in response; Tau = Time constant.
In eq 3.1 time constant can be expressed as,

$$\begin{cases} Tau = \frac{TD-x}{\ln\left(\frac{A+Yb-y}{A}\right)}, (x \neq TD \text{ and } y \neq Yb \text{ and } y > Yb + A \text{ and } A < 0) \text{ or} \\ \qquad (x \neq TD \text{ and } y \neq Yb \text{ and } y < Yb + A \text{ and } A > 0) \\ t \neq 0, \qquad y = Yb \text{ and } (A = 0 \text{ or } x = TD) \end{cases} \qquad \text{.... eq 3.2}$$

Note that, this is just the solve for Tau from Eq 3.1, actual values are calculated by the software (Origin Pro function ExpAssocDelay1.FDF)

The function used for fitting thylakoid samples measurements,

$$y = y_0 + A_1\, e^{-\frac{X-x_0}{t_1}} \qquad \text{.... eq 4.1}$$

where y_0 = Offset, x_0 = Center, A_1 = Amplitude, t_1 = Time constant.
In eq 4.1 time constant can be expressed as,

$$t1 = \begin{cases} \frac{x0-x}{ln\left(\frac{y-y0}{A1}\right)}, x \neq x0 \text{ and } y \neq y1 + A \text{ and } y \neq y0 \text{ and } (y < y0 \text{ or } A1 > 0) \text{ and} \\ \qquad (A1 < 0 \text{ or } y > y0) \text{ and } A1 \neq 0 \\ t1 \neq 0, \quad (y = y1 \text{ and } A1 = 0) \text{or } (y \neq y0 + A1 \text{ and } x = x0 \text{ and } A1 \neq 0) \end{cases} \qquad \text{.... eq 4.2}$$

Note that, this is just the solve for Tau from Eq 4.1, actual values are calculated within the software (Origin Pro function FITFUNC\EXPDECY1.FDF)

Source data are provided as a Source Data file.

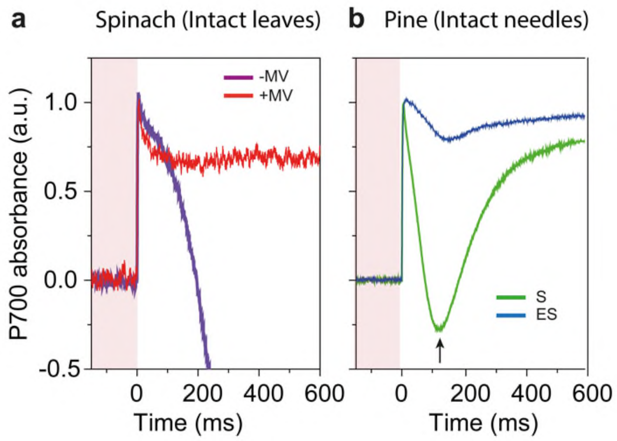

Supplementary Figure 6: Saturating pulse (SP) induced changes in P700 absorbance in intact pine needles. P700 absorbance was recorded on a FR light background (Red shaded region) with a 600ms SP of 4000 µmol of photons m^{2}- s^{-1} in **(a)** spinach (n=3) and **(b)** pine needles (n=3). Spinach leaves and summer pine (S) needles were also vacuum infiltrated with 50µM methyl viologen. (A) In Spinach, upon switching on SP P700 absorbance sharply increases and reaches maximum oxidation level within 2-3ms. Then P700 signal dropped for the rest of SP period following biphasic kinetics because of reduction of P700 via electrons supplied from the luminal components. When MV was vacuum infiltrated prior to measurement, upon switching on SP, P700 dropped only by 15-20% and remained stable for the rest of the SP period. This indicated after maximum oxidation of P700 with SP, even though electrons were supplied from the luminal side but MV being a strong electron acceptor, consumed those electrons rapidly from the acceptor-side of PSI and thereby leaving P700 in an oxidized state. n=number of measurements where needles/thylakoids were pulled from 3-5 biological replicates. **(b)** In S needles P700 reached maximum oxidation levels within 2-3ms, and then started dropping as a result incoming of electrons from the luminal side, but approximately after 100ms become re-oxidized (black arrow) and almost reached the maximum level at the end of the 600ms SP period. This typical characteristics of P700 under strong illumination is well characterized as a consequence of electron consumption by flavodiiron proteins in microalgae and cyanobacteria. In early spring needles (ES), the P700 signal behaved almost like MV treated spinach leaves. P700 signal only dropped by 12-15% and then went back to the maximum level. This might be interpreted as lack of electron flow from the luminal side, but as PQ is heavily reduced, therefore, this P700 characteristic strongly indicated that the electrons were provide to P700 from the reduced PQ pool but were immediately taken up by flavodiiron proteins that in turn reduced O_2 to H_2O. Source data are provided as a Source Data file.

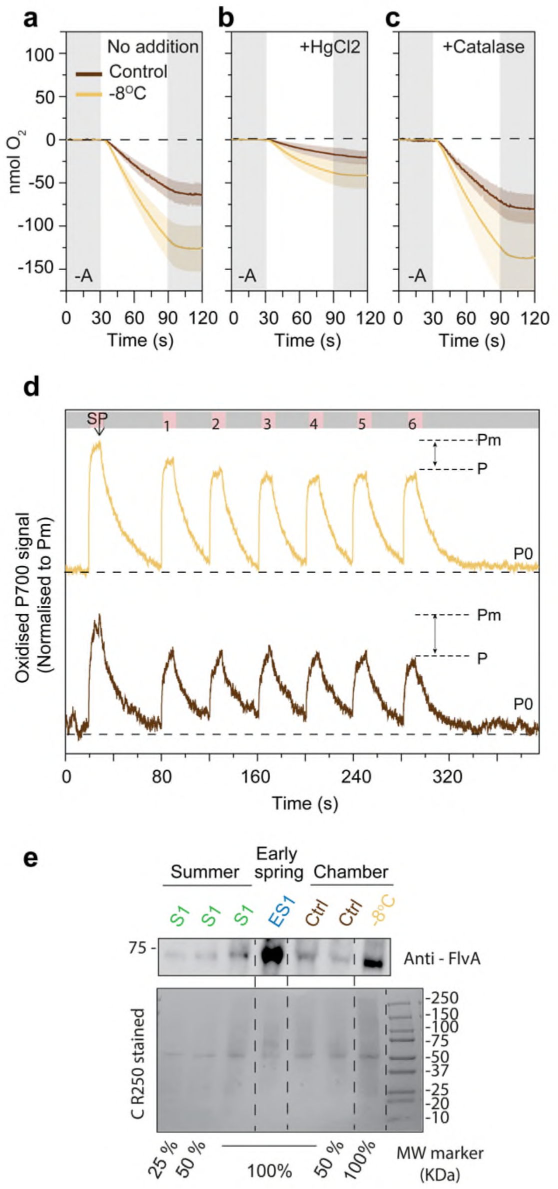

Supplementary Figure 7: Acclimation of pine tree in low temperature in climate chamber. (a) O_2 yields in Control and sub-zero (-8°C) acclimated pine thylakoid membranes in air saturated. **(b)** O_2 yields in control and -8°C thylakoid membranes in air-saturated buffer measured by a Clark-electrode with 1 mg ml^{-1} $HgCl_2$ supplementation. **(c)** O_2 yields in control and -8°C thylakoid membranes in air-saturated buffer measured by a Clark-electrode with 1000-unit ml^{-1} catalase supplementation. All Clark-electrode measurements of O_2 in **(a)**, **(b)** and **(c)** were performed in absence of buffer in absence of exogenous electron acceptors, PPBQ and FeCy (-A). The colored shaded region following the O_2 yield curve (mean) indicates ±SEM (n=3). In all gas exchange, data represents O_2 exchange corresponding to the same chlorophyll content (50 µg). **(d)** Changes in P700 absorbance in control and -8°C pine thylakoid membranes (corresponding to 100 µg/ml chlorophyll) measured with 6 cycles of intermittent FR illumination in the presence of 30 µM antimycin A (*n*=3). **(e)** Relative abundance of flavodiiron A protein in S, ES, control (Ctrl) and -8°C pine thylakoid membranes. Summer sample S1 and early spring sample ES1, control and -8°C sample corresponding to 4 µg of chlorophyll (100%) were loaded in separate lanes and 1 µg and 2 µg of chlorophyll (25% and 50%) of S1 were loaded in the left two lanes and 50% of control sample was loaded on second lane (from right) as quality controls, and the gel was immunoblotted against anti-FlvA antibody. A Coomassie stained membrane is shown in the bottom panel. Similar immunoblotting results were obtained in four independent experiments. In all experiments n=number of biological replicate groups, where one group comprised of thylakoids pulled from 4 plants

in control and 5 plants in -8°C conditions, hence, total 12 plants pulled into 3 groups (i.e., n=3) in control and 15 plants pulled into 3 groups (i.e., n=3) in -8°C conditions. Source data are provided as a Source Data file.

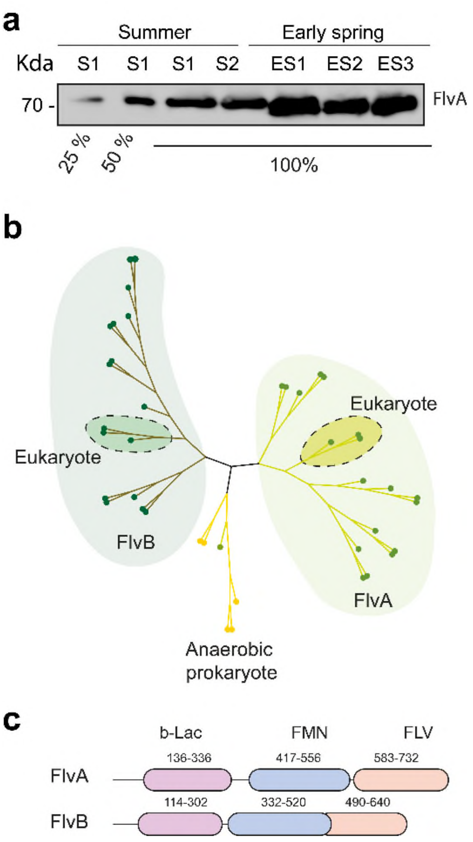

Supplementary Figure 8: Expression and predicted domains of flavodiiron proteins in spruce. (a) Relative abundance of flavodiiron A protein in S and ES thylakoid membranes. Two summer samples (S1, S2) and three early spring samples (ES1, ES2, ES3) (See *Supplementary Table. S1* for sample ID details) corresponding to 4 µg of chlorophyll (100%) were loaded in each lane along with 1 µg and 2 µg of chlorophyll (25% and 50%) as quality control in the left two lanes and immunoblotted with Anti-FlvA antibody. For relative quantitation of S and ES see *Supplementary Table. S4*. Similar immunoblotting results were obtained in four independent experiments. **(b)** Phylogenetic analysis of FlvA and FlvB predicted protein sequences with other flavodiiron and flavodiiron-like proteins from different species {*Prochlorococcus marinus MIT9211, Prochlorococcus marinus* str AS9601, *Prochlorococcus marinus* str MIT9215, *Prochlorococcus marinus* str MIT9312, *Synechococcus elongatus* PCC6301, *Synechococcus sp* WH7803, *Synechococcus sp* CC9605, *Synechococcus elongatus* PCC7942, *Trichodesmium erythraeum* IMS101, *Physcomitrella_patens, Chlamydomonas_reinhardtii, Synechocystis sp* PCC6803, *Nostoc sp* PCC7120, *Arthrospira_platensis* NIES-39, *Cyanothece sp* ATCC51142, *Thermosynechococcus elongatus* BP-1, *Chlorobium tepidum* TLS, *Desulfovibrio gigas, Escherichia_coli* str K-12, *Moorella_thermoacetica,* and *Picea_abies*} (See *Supplementary dataset 1* for list of the Flv protein sequences in other organisms). In the unrooted tree, nodes indicate different Flv proteins in different species and branches indicate their relative distances. Three main branches, yellow, light green and dark green indicate NOR (Nitric oxide reductase) domain containing Flv-like, FlvA and FlvB proteins, respectively. For construction of the tree, default parameters of IQ-TREE have been used, where sequences were identified as proteins, substitutional model was chosen 'auto', field FreeRate heterogeneity was chosen as +I, allowing for a proportion of invariable sites. An ascertainment bias correction (+ASC) was added to prevent over-estimation of branch length. Branch Support Analysis was performed with the following parameters – ultrafast bootstrap mode, number of bootstrap alignments 1000, maximum iterations 1000 and maximum correlation coefficient 0.99. No single branch tests were performed and the perturbation strength for randomized nearest neighbor interchange was chosen 0.5. **(c)** Domain prediction of spruce FlvA and FlvB proteins. Both FlvA and FlvB contain all three characteristic domains, namely, beta-lactamase domain (b-Lac), flavine

mono nucleotide domain (FMN) and flavodiiron domain (FLV). Source data are provided as a Source Data file.

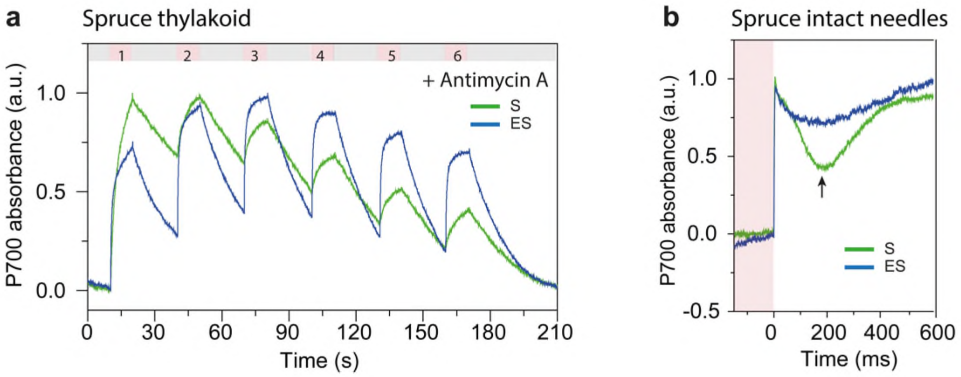

Supplementary Figure 9: P700 oxidation kinetics in spruce measured with intermittent FR on thylakoid and with saturating pulse (SP) method on intact needles. (a) Changes in P700 oxidation in spruce thylakoid were recorded (n=3) by providing consecutive 6 cycles of 10s FR illumination followed by 30s darkness. FR light intensity was 250 µmol of photons m^{-2} s^{-1}. Before measurement thylakoid samples were incubated with 30µM Antimycin A, a known CEF blocker. **(b)** P700 absorbance changes in intact spruce needles by SP were recorded (n=3) as shown in *Supplementary Fig 5* for pine needles**.** Note that S needles had lower amplitude of P700 reduction after max levels are reached within 2-3ms of SP application compared to S pine needles. Nevertheless, ES needles behaved similar but not identical to ES pine needles. n=number of measurements where needles/thylakoids were pulled from 3-5 biological replicates. Source data are provided as a Source Data file.

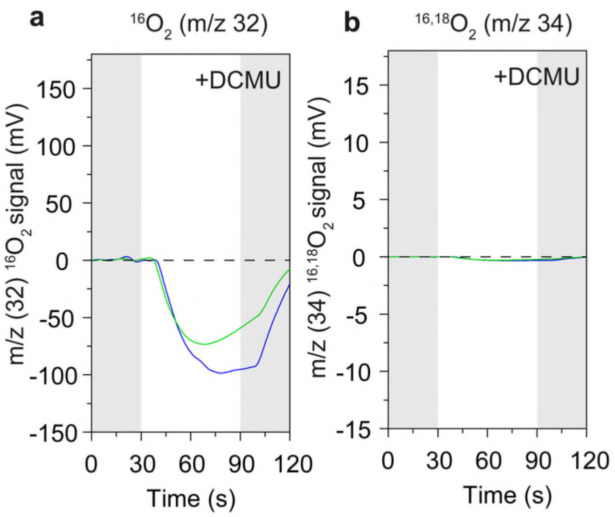

Supplementary Figure 10: (a) $^{16}O_2$ and (b) $^{16,18}O_2$ yields in S and ES spruce thylakoid membranes in air saturated buffer with 250 µM PPBQ and 500 µM FeCy supplemented with 25 µM DCMU measured with TR-MIMS in partially degassed condition. Grey shaded region indicates dark period before and after illumination of the thylakoid membranes with 1200 µmol of photons m^{-2} s^{-1} for 60 s. In all gas exchange experiments data represent O_2 exchange corresponding to 50 µg of chlorophyll. (For data analysis details see Supplementary Fig S2). For TR-MIMS one representative trace out of 3-5 independent measurements is shown. Source data are provided as a Source Data file.

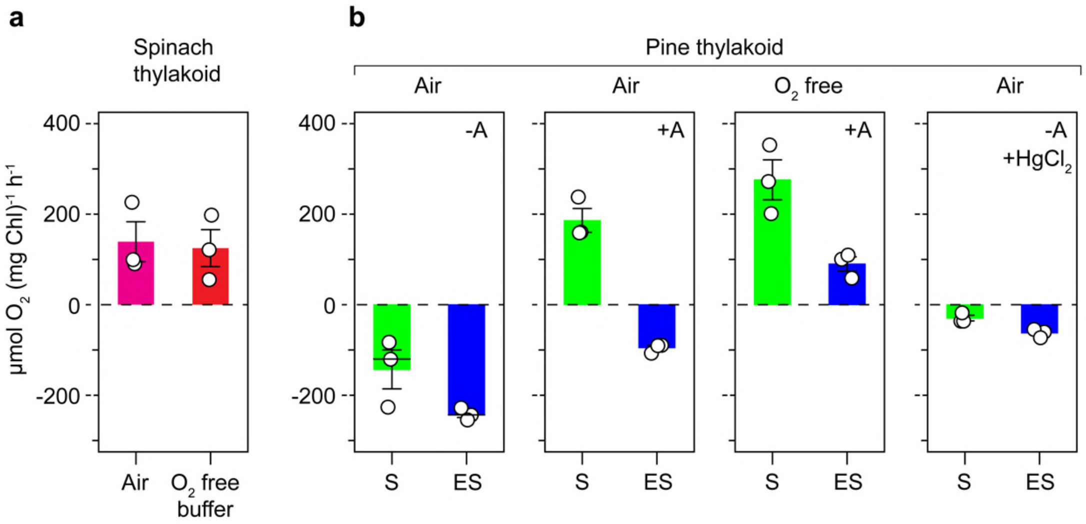

Supplementary Figure 11: Rates of O_2 exchange in terms of µmol of O_2 (mg of Chlorophyll)$^{-1}$ h^{-1}. Rates of O_2 exchange in **(a)** Spinach and (**b)** Pine thylakoid membranes in S and ES. Rates were calculated as linear function of time ($\boldsymbol{y} = \boldsymbol{m} * \boldsymbol{x} + \boldsymbol{c}$); where x is the slope from O_2 the exchange during the initial (10 s) rise or decrease of the O_2 signal induced by the illumination. The rate of O_2 evolution is maximum at this stage and the overall O_2 exchange yields were more dependent on O_2 consumption in the later stages of illumination. Here -A and +A indicates absence and presence, respectively, of the exogenous electron acceptor 250 µM PPBQ and 500 µM FeCy. Bars indicate the average of three independent measurements that are represented with open circles. Error bars indicate the ±SEM (n=3); n=biological replicates. Note that these rates are 20-25 times higher than the rates reported in intact thylakoid membranes due to the presence of exogenous electron acceptor and the absence of a proton gradient due leakiness of the membranes. This way, we were able to quantify the O_2 consumption and PSII O_2 evolution simultaneously. No statistics were performed as rates are derived parameters and not measured directly. Source data are provided as a Source Data file.

Supplementary References:

[1] A. Savitzky and M. J. E. Golay, "Smoothing and differentiation of data by simplified least squares procedures.," *Anal. Chem.*, vol. 36, no. 8, pp. 1627–1639, 1964.

[2] A. W. Rutherford, A. Osyczka, and F. Rappaport, "Back-reactions, short-circuits, leaks and other energy wasteful reactions in biological electron transfer: redox tuning to survive life in O2," *FEBS Lett.*, vol. 586, no. 5, pp. 603–616, 2012.

[3] C. Fufezan, C. M. Gross, M. Sjodin, A. W. Rutherford, A. Krieger-Liszkay, and D. Kirilovsky, "Influence of the redox potential of the primary quinone electron acceptor on photoinhibition in photosystem II," *J. Biol. Chem.*, vol. 282, no. 17, pp. 12492–12502, 2007.

[4] A. W. Rutherford and A. Krieger-Liszkay, "Herbicide-induced oxidative stress in photosystem II," *Trends Biochem. Sci.*, vol. 26, no. 11, pp. 648–653, 2001.

[5] S. Khorobrykh, V. Havurinne, H. Mattila, and E. Tyystjärvi, "Oxygen and ROS in Photosynthesis," *Plants*, vol. 9, no. 1, p. 91, 2020.

[6] W. H. J. Wood *et al.*, "Dynamic thylakoid stacking regulates the balance between linear and cyclic photosynthetic electron transfer," *Nat. Plants*, vol. 4, no. 2, pp. 116–127, 2018.
