# Supplementary material for: Flavodiiron-mediated O2 photoreduction at photosystem I acceptor-side provides photoprotection to conifer thylakoids in early spring
Source: Nat Commun. 2023 Jun 3;14:3210. doi: 10.1038/s41467-023-38938-z (PMC10239515; doi:10.1038/s41467-023-38938-z)
Supplement: Supplementary file 2 — Reporting Summary [file 41467_2023_38938_MOESM2_ESM.pdf]

nature portfolio

Corresponding author(s): Stefan Jansson

Last updated by author(s): May 9, 2023

Reporting Summary

Nature Portfolio wishes to improve the reproducibility of the work that we publish. This form provides structure for consistency and transparency in reporting. For further information on Nature Portfolio policies, see our Editorial Policies and the Editorial Policy Checklist.

Statistics

For all statistical analyses, confirm that the following items are present in the figure legend, table legend, main text, or Methods section.

n/a	Confirmed	
☐	☒	The exact sample size (*n*) for each experimental group/condition, given as a discrete number and unit of measurement
☐	☒	A statement on whether measurements were taken from distinct samples or whether the same sample was measured repeatedly
☐	☒	The statistical test(s) used AND whether they are one- or two-sided<br>*Only common tests should be described solely by name; describe more complex techniques in the Methods section.*
☒	☐	A description of all covariates tested
☒	☐	A description of any assumptions or corrections, such as tests of normality and adjustment for multiple comparisons
☐	☒	A full description of the statistical parameters including central tendency (e.g. means) or other basic estimates (e.g. regression coefficient) AND variation (e.g. standard deviation) or associated estimates of uncertainty (e.g. confidence intervals)
☐	☒	For null hypothesis testing, the test statistic (e.g. *F*, *t*, *r*) with confidence intervals, effect sizes, degrees of freedom and *P* value noted<br>*Give P values as exact values whenever suitable.*
☒	☐	For Bayesian analysis, information on the choice of priors and Markov chain Monte Carlo settings
☒	☐	For hierarchical and complex designs, identification of the appropriate level for tests and full reporting of outcomes
☒	☐	Estimates of effect sizes (e.g. Cohen's *d*, Pearson's *r*), indicating how they were calculated

Our web collection on statistics for biologists contains articles on many of the points above.

Software and code

Policy information about availability of computer code

Data collection	Oxygraph+, Dual PAM 100 V3.19, Mass Hunter Profinder version B.08.00
Data analysis	Mass Hunter Profinder (version B.08.00), Origin Pro 2018b (Version 9.55) , Origin Pro 2021 (Version 9.95), Origin Pro 2022 (Version 9.9), SPSS (Version 27), ImageJ (Version 1.53t)

For manuscripts utilizing custom algorithms or software that are central to the research but not yet described in published literature, software must be made available to editors and reviewers. We strongly encourage code deposition in a community repository (e.g. GitHub). See the Nature Portfolio guidelines for submitting code & software for further information.

Data

Policy information about availability of data

All manuscripts must include a data availability statement. This statement should provide the following information, where applicable:

- Accession codes, unique identifiers, or web links for publicly available datasets
- A description of any restrictions on data availability
- For clinical datasets or third party data, please ensure that the statement adheres to our policy

All raw and source data for figure 1-3 and supplementary figure 1-11 are provided in the source data file. Peptide sequences for phylogenetic analysis are provided in supplementary dataset 1 and statistical details for supplementary table 4 are provided in supplementary dataset 2. All steps of data analysis to reach the source data from the original raw data are provided in the pipeline in the source data file.

Research involving human participants, their data, or biological material

Policy information about studies with human participants or human data. See also policy information about sex, gender (identity/presentation), and sexual orientation and race, ethnicity and racism.

Reporting on sex and gender	Not applicable
Reporting on race, ethnicity, or other socially relevant groupings	Not applicable
Population characteristics	Not applicable
Recruitment	Not applicable
Ethics oversight	Not applicable

Note that full information on the approval of the study protocol must also be provided in the manuscript.

Field-specific reporting

Please select the one below that is the best fit for your research. If you are not sure, read the appropriate sections before making your selection.

☒ Life sciences ☐ Behavioural & social sciences ☐ Ecological, evolutionary & environmental sciences

For a reference copy of the document with all sections, see nature.com/documents/nr-reporting-summary-flat.pdf

Life sciences study design

All studies must disclose on these points even when the disclosure is negative.

Sample size	For outdoor experiments samples were drawn from 3-5 mature pine and spruce trees. All gas exchange measurements on thylakoids were performed with biological replicates (n=3) and for intact needle measurements needles were pulled from the five trees (n=5) and then subjected to 2-3 independent measurements. No technical measurements were performed on the same sample. For climate chamber experiments thylakoids pulled in three groups comprised of 4 plants in control and 5 plants in -8°C conditions, hence, total 12 plants pulled into 3 groups (i.e., n=3) in control and 15 plants pulled into 3 groups (i.e., n=3) in -8°C conditions. Specific n is given with each figure/table legend. These replications were sufficient for calculating Mean, SD and SEM.
Data exclusions	No data has been excluded from the study samples that has been highlighted in yellow in supplementary table 1. Only one spruce blot was not considered for quantization as the image had higher background black values and the software was not precise to separate the background intensity from the protein intensity. However, visual identification of bands was still possible, therefore, this blot is still shown with the raw data (Third blot spruce section).
Replication	All experiments were replicated two-five times independent of biological replication. This was either done by performing the measurements on different dates during the same season or by by repeating the experiment with different batch of samples on the same day.
Randomization	All needle samples were drawn from random branches of the spruce and pine trees
Blinding	Not applicable

Reporting for specific materials, systems and methods

We require information from authors about some types of materials, experimental systems and methods used in many studies. Here, indicate whether each material, system or method listed is relevant to your study. If you are not sure if a list item applies to your research, read the appropriate section before selecting a response.

Materials & experimental systems

n/a	Involved in the study	
☐	☒	Antibodies
☒	☐	Eukaryotic cell lines
☒	☐	Palaeontology and archaeology
☒	☐	Animals and other organisms
☒	☐	Clinical data
☒	☐	Dual use research of concern
☐	☒	Plants

Methods

n/a	Involved in the study	
☒	☐	ChIP-seq
☒	☐	Flow cytometry
☒	☐	MRI-based neuroimaging

Antibodies

Antibodies used	Primary antibodies recognizing FlvA proteins in Norway spruce (Named Anti-FlvA here after) was provided by Professor Eva-Mari Aro, Turku university, Finland. The antibody was raised in rabbit, against a peptide derived from the Norway spruce FlvA sequence and used in 1:2000 dilution. Secondary anti-rabbit antibody (AgriseraAB, Vännäs, Sweden - AS101461) was used at 1:15000 dilution. Information can be provided on the manufacturer's website.
Validation	Initial validation of anti-FlvA was done in Prof. Aro's lab by western blotting against Norway spruce thylakoid membranes. Later we have validated the Anti-FlvA antibody in both Scots Pine and Norway spruce by western blotting against the thylakoid membranes. We have standardized the optimal antibody activity by using 2 different dilutions (1:2000 and 1:5000) in western blotting and used in 1:2000 dilution for the study.

Dual use research of concern

Policy information about dual use research of concern

Hazards

Could the accidental, deliberate or reckless misuse of agents or technologies generated in the work, or the application of information presented in the manuscript, pose a threat to:

No	Yes	
☒	☐	Public health
☒	☐	National security
☒	☐	Crops and/or livestock
☒	☐	Ecosystems
☒	☐	Any other significant area

Experiments of concern

Does the work involve any of these experiments of concern:

No	Yes	
☒	☐	Demonstrate how to render a vaccine ineffective
☒	☐	Confer resistance to therapeutically useful antibiotics or antiviral agents
☒	☐	Enhance the virulence of a pathogen or render a nonpathogen virulent
☒	☐	Increase transmissibility of a pathogen
☒	☐	Alter the host range of a pathogen
☒	☐	Enable evasion of diagnostic/detection modalities
☒	☐	Enable the weaponization of a biological agent or toxin
☒	☐	Any other potentially harmful combination of experiments and agents
