# Supplementary material for: Flavodiiron-mediated O2 photoreduction at photosystem I acceptor-side provides photoprotection to conifer thylakoids in early spring
Source: Nat Commun. 2023 Jun 3;14:3210. doi: 10.1038/s41467-023-38938-z (PMC10239515; doi:10.1038/s41467-023-38938-z)
Supplement: Supplementary file 3 — Description of Additional Supplementary Files [file 41467_2023_38938_MOESM3_ESM.pdf]

Title: Supplementary data 1.
Description: Fasta sequences for phylogenetic analysis of FlvA and FlvB predicted protein sequences with other flavodiiron and flavodiiron-like proteins from different species {Prochlorococcus marinus MIT9211, Prochlorococcus marinus str AS9601, Prochlorococcus marinus str MIT9215, Prochlorococcus marinus str MIT9312, Synechococcus elongatus PCC6301, Synechococcus sp WH7803, Synechococcus sp CC9605, Synechococcus elongatus PCC7942, Trichodesmium erythraeum IMS101, Physcomitrella_patens, Chlamydomonas_reinhardtii, Synechocystis sp PCC6803, Nostoc sp PCC7120, Arthrospira_platensis NIES-39, Cyanothece sp ATCC51142, Thermosynechococcus elongatus BP-1, Chlorobium tepidum TLS, Desulfovibrio gigas, Escherichia_coli str K-12, Moorella_thermoacetica, and Picea_abies}.

Title: Supplementary data 2.
Description: Detailed statistical analysis for supplementary table 5. Sheet 1 contains normalised black values of the original blots (Raw data provided in folder **Fig 3 suppl 8/fig 3f supple 8a**). Sheet 2 contains statistical details for pine and sheet 3 contains details for spruce (not that 3rd spruce blot in the raw data folder is not included in quantification).
