# Supplementary material for: Flavodiiron-mediated O2 photoreduction at photosystem I acceptor-side provides photoprotection to conifer thylakoids in early spring
Source: Nat Commun. 2023 Jun 3;14:3210. doi: 10.1038/s41467-023-38938-z (PMC10239515; doi:10.1038/s41467-023-38938-z)
Supplement: Supplementary file 7 — Source Data [file 41467_2023_38938_MOESM7_ESM.zip › Raw data files/Fig 3 suppl 8/Fig 3f supple 8a/All blot replicates and gel images.pdf]

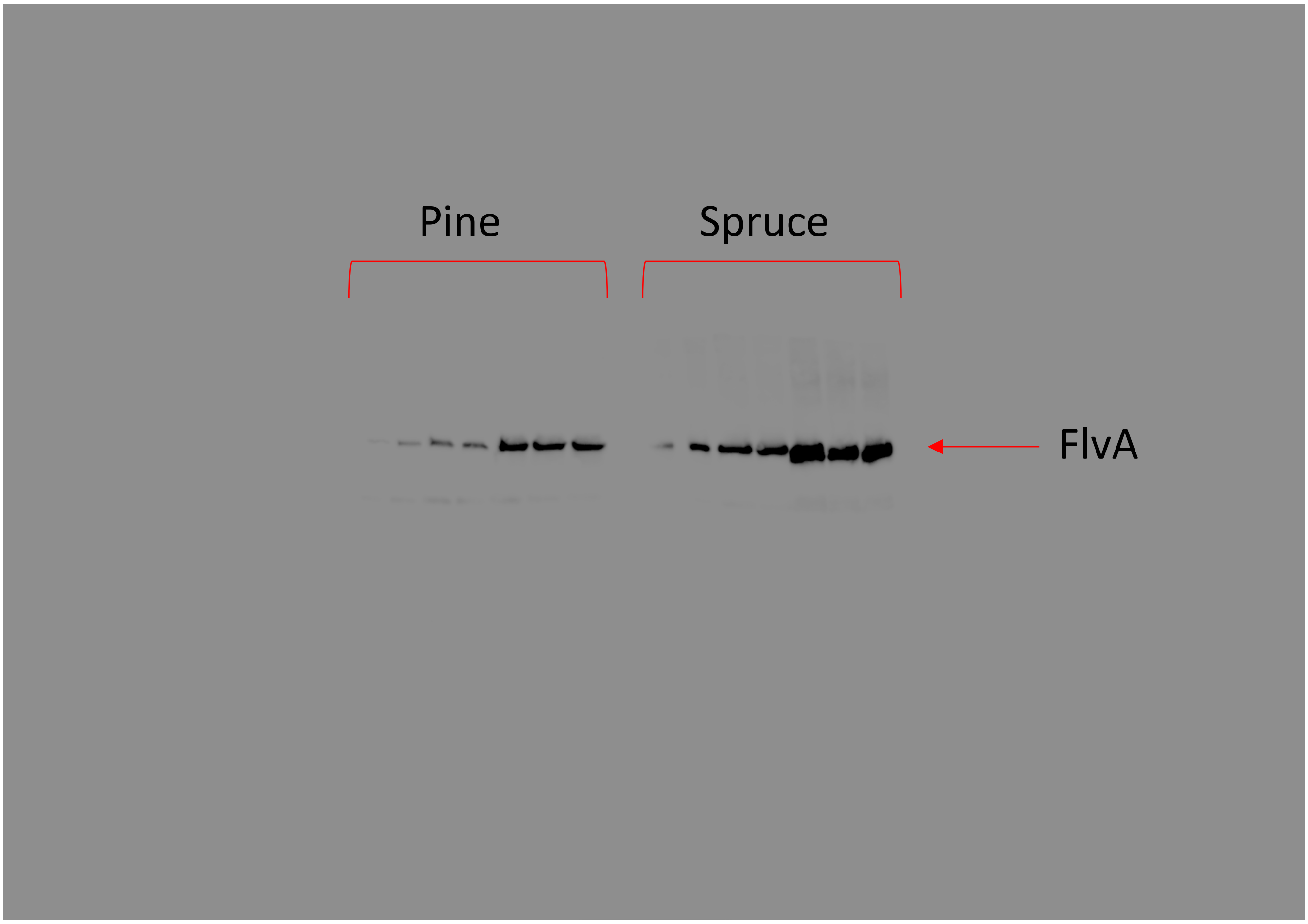


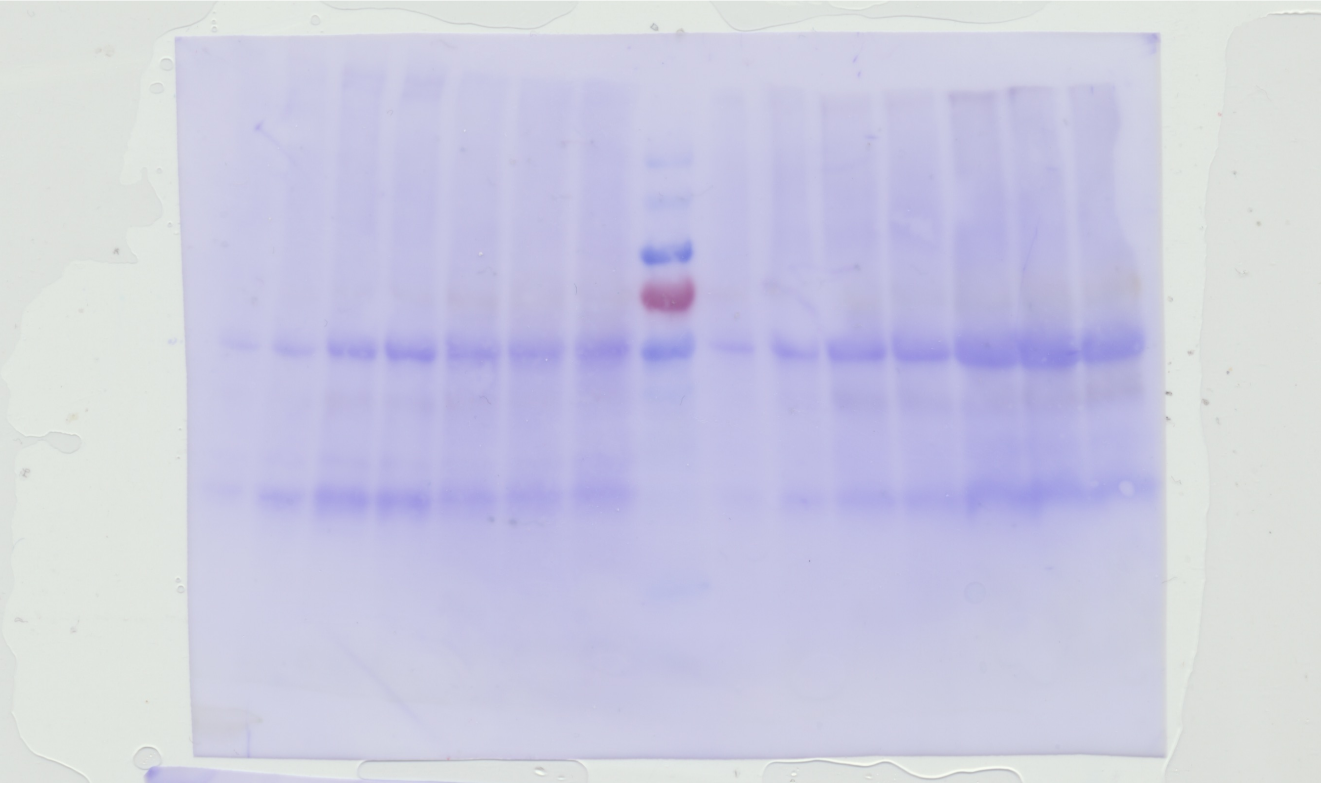

Membrane in RGB scale

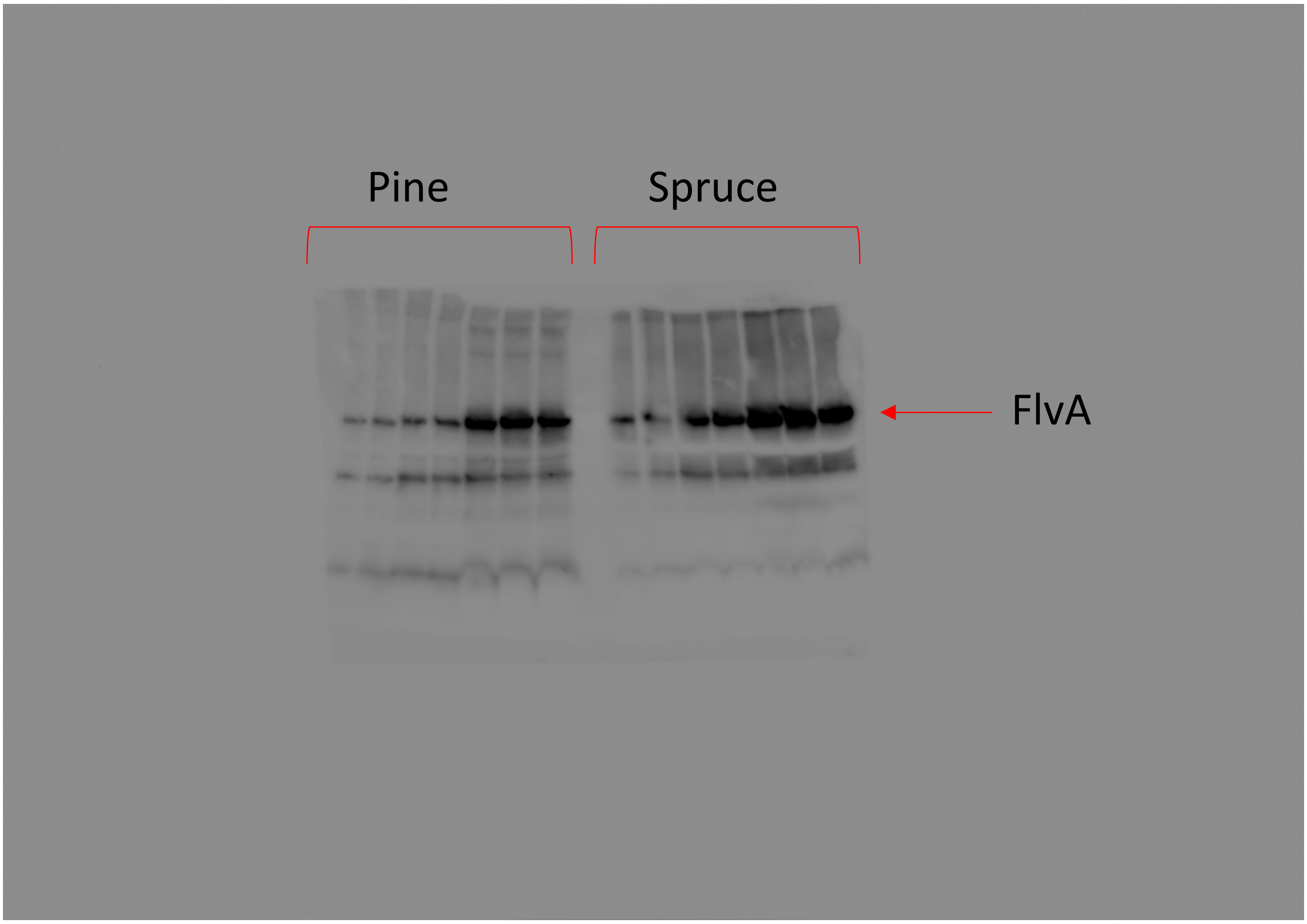


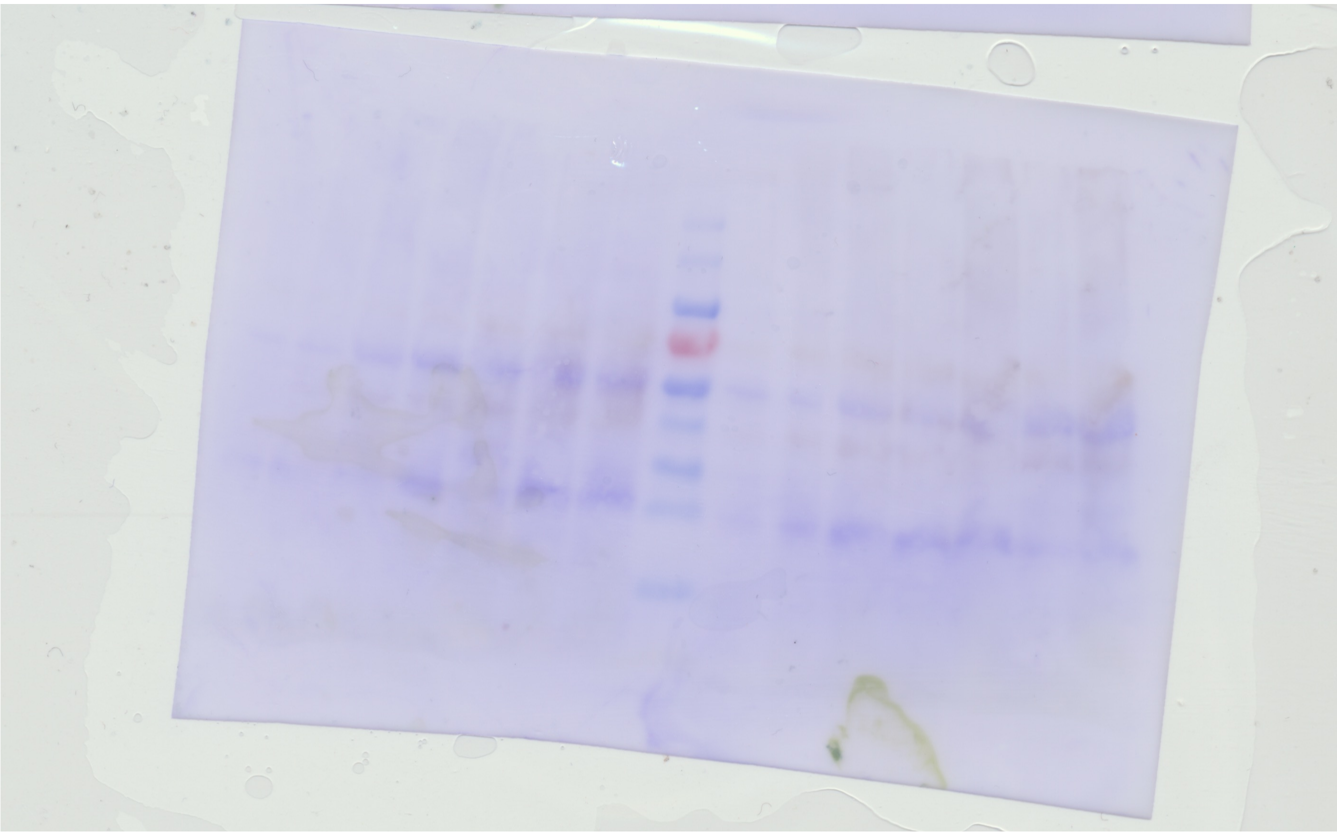

Membrane in RGB scale

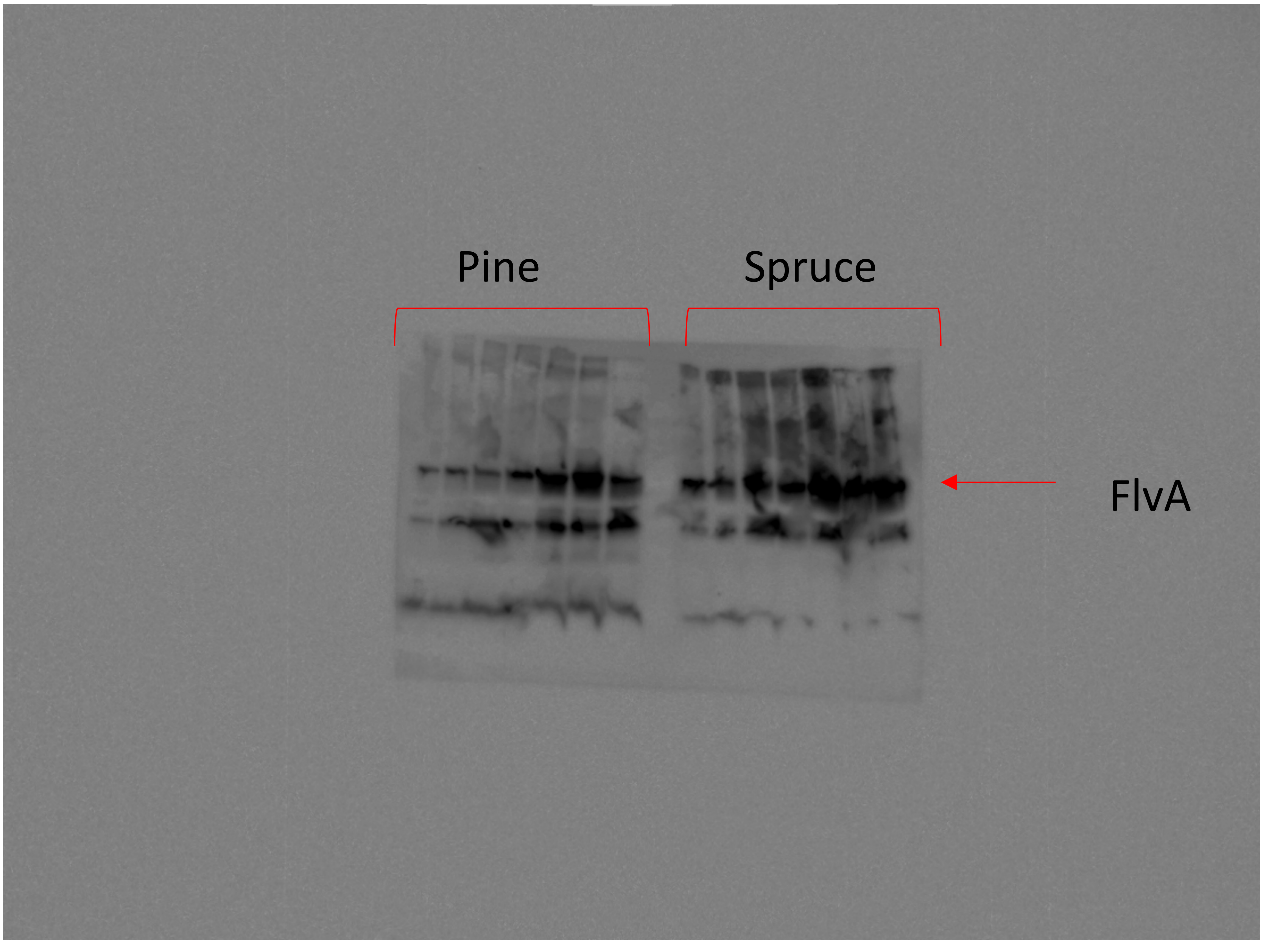


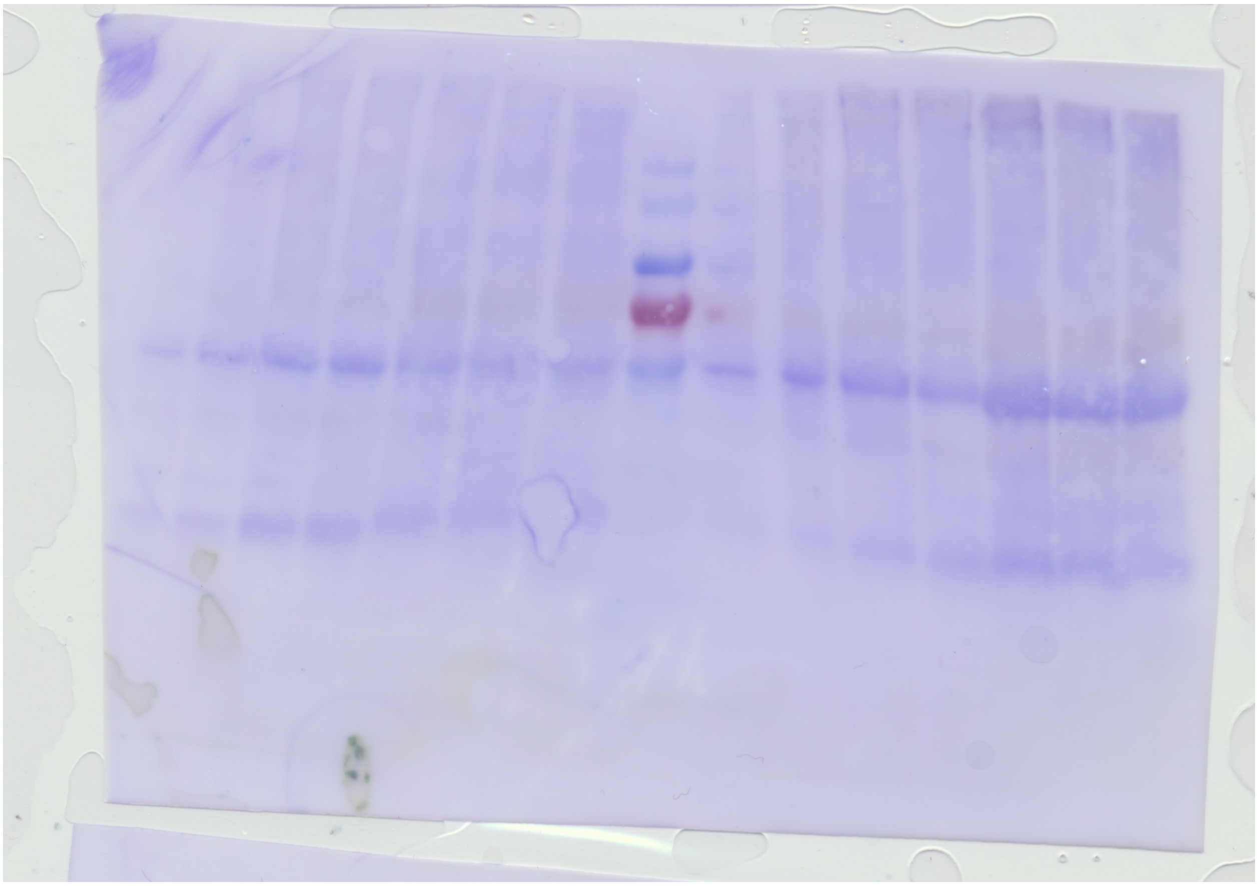

Membrane in RGB scale

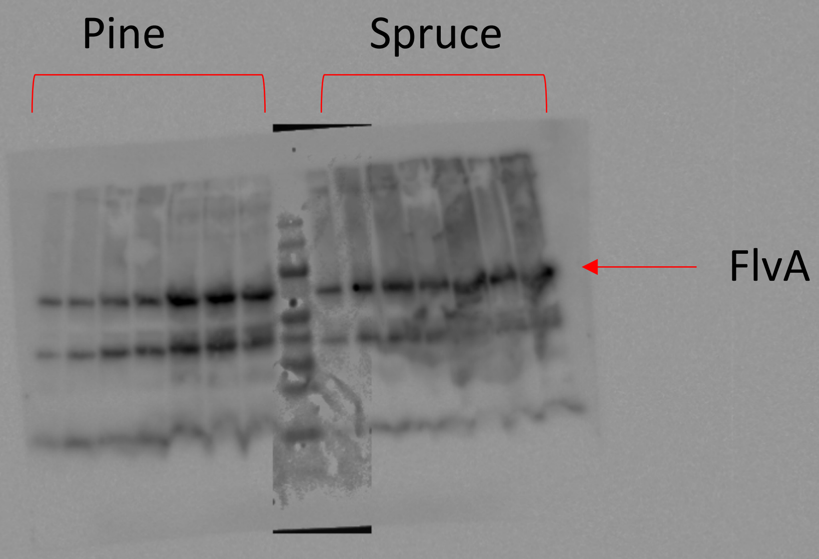


This image has both chemiluminescent and RBG marker region overlap, generated withn the AZURE imaging system for comparision of ladder in chemiluminescent and RBB codition

Membrane was not scanned RGB scale
