# Supplementary material for: Flavodiiron-mediated O2 photoreduction at photosystem I acceptor-side provides photoprotection to conifer thylakoids in early spring
Source: Nat Commun. 2023 Jun 3;14:3210. doi: 10.1038/s41467-023-38938-z (PMC10239515; doi:10.1038/s41467-023-38938-z)

Thermofisher ladder

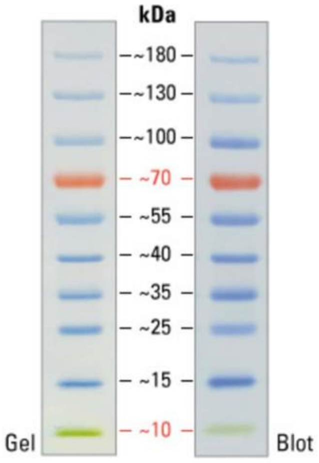

Thermofisher ladder on the membrane used for Flv blots
(Membrane stained with Comassie, since gel was not stained)

Thermofisher ladder on the membrane used for Flv blots
(Membrane scan before chemiluminiscent reagent added)

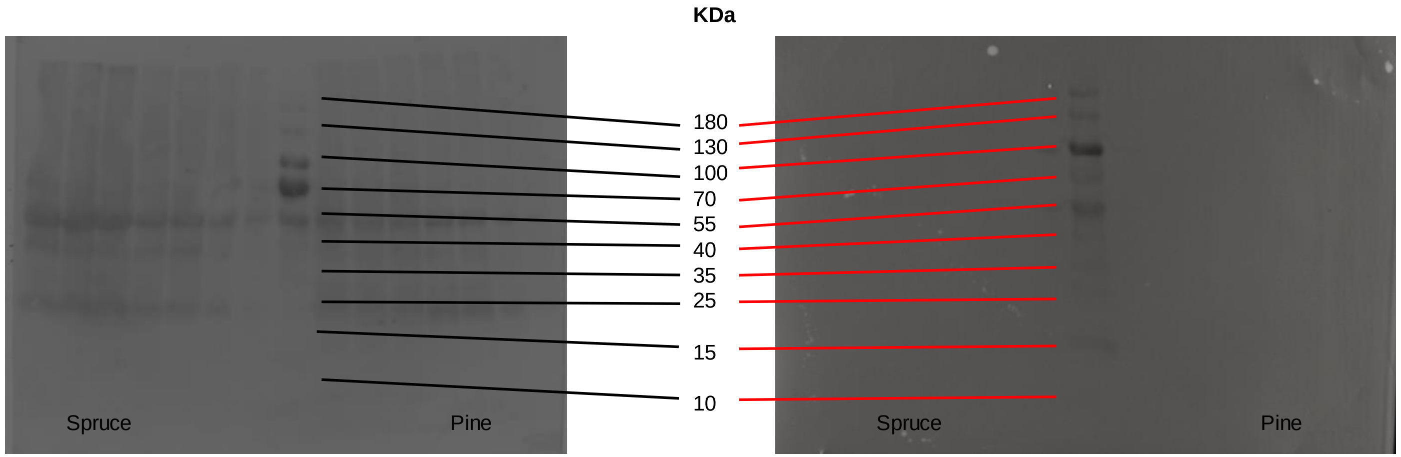

Ladder + Flv blots overlay
(Membrane scan after chemiluminiscent reagent added and excited)

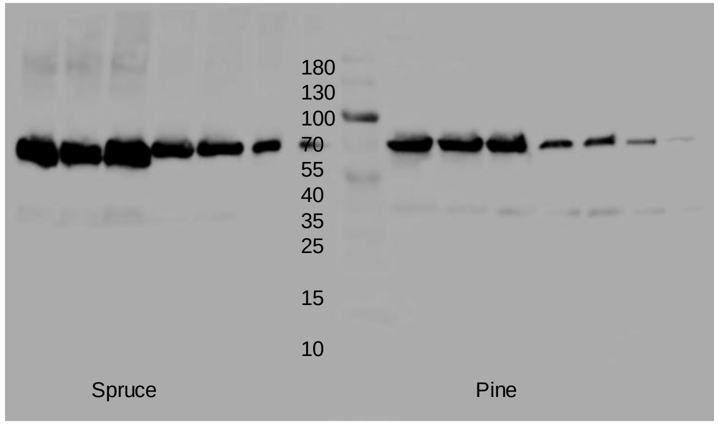

Supplement: Supplementary file 7 — Source Data [file 41467_2023_38938_MOESM7_ESM.zip › Raw data files/Fig 3 suppl 8/Fig 3f supple 8a/Ladder and band identification.pdf]
