# Supplementary material for: Flavodiiron-mediated O2 photoreduction at photosystem I acceptor-side provides photoprotection to conifer thylakoids in early spring
Source: Nat Commun. 2023 Jun 3;14:3210. doi: 10.1038/s41467-023-38938-z (PMC10239515; doi:10.1038/s41467-023-38938-z)
Supplement: Supplementary file 7 — Source Data [file 41467_2023_38938_MOESM7_ESM.zip › Raw data files/Pipeline.pdf]

This file contains information for the data analysis pipelines that has been used in Origin Pro and SPSS for analysing O_2 traces, Fluorescence and P700 traces and statistical analysis in the following manuscript.

Flavodiiron-mediated O_2 photoreduction at photosystem I acceptor-side provides photoprotection to conifer thylakoids in early spring

Authors: Pushan Bag[1, 6,], Tatyana Shutova[1, 6], Dmitry Shevela[2], Jenna Lihavainen[1], Sanchali Nanda[1], Alexander G. Ivanov[3, 4], Johannes Messinger[2, 5], Stefan Jansson[1, *]

Affiliations: [1]Umeå Plant Science Centre, Department of Plant Physiology, Umeå University, Umeå, Sweden, [2]Department of Chemistry, Chemical Biological Centre, Umeå University, Umeå, Sweden, [3]Department of Biology, University of Western Ontario, London, Ontario, Canada, [4]Institute of Biophysics and Biomedical Engineering, Bulgarian Academy of Sciences, Sofia, Bulgaria, [5]Department of Chemistry – Ångström laboratory, Uppsala University, Uppsala, Sweden
[6]These authors contributed equally to this work
*Corresponding author (stefan.jansson@umu.se)

Author contribution: TS, PB and SJ conceived the idea; PB, TS, DS, JL, AGI, JM, and SJ designed the research; PB and TS performed Clarke-electrode measurements, TS and DS performed MIMS measurements, JL performed the prenyl pool measurements, PB and AGI performed P700 measurements, PB and SN performed immunodetection; JM and SJ contributed to reagents, tools and supervised experiments; PB, TS, DS, AGI, JL, JM and SJ analyzed the data; PB, JM and SJ wrote the paper with input from all co-authors.

Authors' OCRID:

PB: orcid.org/0000-0003-3858-4606
TS: orcid.org/0000-0002-4095-9609
DS: orcid.org/0000-0002-5174-083x
JL: orcid.org/0000-0001-7979-8876
SN: orcid.org/0000-0002-6694-7235
AGI: orcid.org/0000-0001-7100-9211
JM: orcid.org/0000-0003-2790-7721
SJ: orcid.org/0000-0002-7906-6891

This file contains,
Pipelines 1-5

Pipeline 1: Clarke electrode data analysis (All Clarke electrode figures)

In this pipeline, the data processing/analysis of the Clarke-electrode recordings are detailed in step by step.

1. Baseline correction of the raw data

a) Raw data (In respective excel files under columns named as 'Raw') plotted in Origin as line graph

b) Then the following steps were performed

i. Mathematics

ii. peak and baseline

iii. create baseline

iv. user defined

v. 1st and 2nd order derivatives (zeros)(Rest of the parameters remains default)

vi. Clear all default data points

vii. Pick data points manually (from initial dark period prior to illumination where the signal is linear)

viii. Extrapolation by straight line ($y = m * x + C$)

ix. Create baseline

c) Baselines can be found in the respective excel files under columns named 'baseline'

d) Baseline subtracted from the raw data (under columns named 'baseline corrected data')

2. Normalization to equal chlorophyll (50 µg of chlorophyll) if different dilutions of chlorophyll was used for different measurements (Dilutions are provided as 'DF X')

3. Normalized data are in columns named 'normalized to 50 ug Chlorophyll'

4. 'X' axis set to '0' 30s prior to light on (columns named 'Time '0' offset')

5. Average of the replicates plotted with standard errors

Pipeline 2: MIMS data analysis (All MIMS figures)

In this pipeline, the data processing/analysis of the MIMS recordings are detailed in step by step.

1. Baseline correction of the raw data

a) Raw data (In respective excel files under columns named as ‘Raw’) plotted in Origin as line graph

b) Then the following steps were performed

i. Mathematics

ii. peak and baseline

iii. create baseline

iv. user defined

v. 1st and 2nd order derivatives (zeros)(Rest of the parameters remains default)

vi. Clear all default data points

vii. Pick data points manually (from dark period prior and after the illumination)

viii. Extrapolation with B-spline

ix. Create baseline

c) Baselines can be found in the respective excel files under columns named ‘baseline’

d) Baseline subtracted from the raw data (under columns named ‘baseline corrected data’)

2. ‘X’ axis set to ’0’ 30s prior to light on (columns named ‘0’ offset)
3. Representative spectrum was plotted

Pipeline 3.1: P700 data analysis - FR intermittent analysis (Fig 3d and Supplementary Fig 7d, 9a)

In this pipeline, the data processing/analysis of the FR intermittent signal from P700 absorbance recordings are detailed in step by step.

1. Baseline correction (Signal drift) of the raw data

a) Raw data (In respective excel files under columns named as 'Raw') plotted in Origin as line graph

b) Then the following steps were performed

i. Mathematics

ii. peak and baseline

iii. create baseline

iv. user defined

v. 1st and 2nd order derivatives (zeros)(Rest of the parameters remains default)

vi. Clear all default data points

vii. Pick data points manually (from dark period prior and after the illumination)

viii. Extrapolation with B-spline

ix. Create baseline

c) Baselines can be found in the respective excel files under columns named 'baseline'

d) Baseline subtracted from the raw data (under columns named 'baseline corrected data')

2. 'X' axis set to '0' 30s prior to light on (columns named '0' offset)
3. If required data was normalized to max P700 signal (columns named as 'Normalized signal')
4. Representative/Average spectrum was plotted

Pipeline 3.2: P700 data analysis - Intersystem e^- analysis (Fig 2c)

In this pipeline, the data processing/analysis of the Intersystem e^- pool from P700 absorbance recordings are detailed in step by step.

1. Baseline correction of the raw data

a) Raw data (In respective excel files under columns named as 'Raw') plotted in Origin as line graph

b) Then the following steps were performed

x. Mathematics

xi. data manipulation

xii. subtract straight line

xiii. manually pick region of interest for signal drift correction

c) Drift corrected data can be found under columns named 'baseline corrected data'

d) Region of single turnover flash (ST) and multi turnover flash (MT) was integrated using integrate tool.

Pipeline 3.3: P700 data analysis - P700 re-reduction kinetics analysis (Supplementary Fig 5a, c and e)

In this pipeline, the data processing/analysis of the P700 re-reduction kinetics signal from P700 absorbance recordings are detailed in step by step.

1. Baseline correction (Signal drift) of the raw data

a) Raw data (In respective excel files under columns named as 'Raw') plotted in Origin as line graph

b) Then the following steps were performed

xiv. Mathematics

xv. peak and baseline

xvi. create baseline

xvii. user defined

xviii. 1st and 2nd order derivatives (zeros)(Rest of the parameters remains default)

xix. Clear all default data points

xx. Pick data points manually (from dark period prior and after the illumination)

xxi. Extrapolation with B-spline

xxii. Create baseline

c) Baselines can be found in the respective excel files under columns named 'baseline'

d) Baseline subtracted from the raw data (under columns named 'baseline corrected data')

2. 'X' axis set to '0' 5s prior to light off (columns named '0' offset)
3. If required data was normalized between 0 to 1 (columns named as 'Normalized signal')
4. Representative/Average spectrum was plotted

Pipeline 4: P700 data analysis - P700 re-reduction kinetics data fitting and time constant determination (Supplementary Fig 5b, d and f)

In this pipeline, the data processing/analysis of the P700 re-reduction kinetics signal from P700 absorbance recordings to determine the time constant (Tau) are detailed in step by step.

1. Data fitting

- ***a)*** Mathematics
- ***b)*** Curve fitting
- ***c)*** Non-linear curve fitting
- ***d)*** Pick function (as mentioned in the figure legend)
- ***e)*** Select range of data to fit
- ***f)*** Perform iterations one by one until X^2 reaches tolerance value and residual falls in the range between -1 and +1.
- ***g)*** Tau value obtained from the fitting results and plotted as whisker-box plot

Pipeline 5: Statistical analysis using SPSS (t-test and One-way ANNOVA)

1. **t-test**

   a. Analyze → Compare Means → Independent Samples T-test

   b. Test variables: PQ, PQH2, PQ/PQH2, UBQ, UBQH2, UBQ/UBQH2

   c. Grouping variable: Group 1 = 1 summer (S)
   Group 2 = 2 early spring (ES)

   d. Confidence interval percentage: 95%

2. **One-way ANOVA**

   a. Analyze → Compare Means → One-Way ANOVA

   b. Dependent list: s1.r1, s1.r2, s1.r3, s1.r4, s2.r1, s2.r2, s2.r3, s2,r4, e1.r1, e1.r2, e1.r3, e1.r4, e2.r1, e2.r2, e2.r3, e2.r4, e3.r1, e3.r2, e3.r3, e3.r4
   (Where r1-r4 is the number of replicates)

   c. Factor: Day (s1, s2, e1, e2, e3)

   d. Post Hoc: LSD

   e. Confidence intervals: 95%
