# Supplementary figures and images for: Flavodiiron-mediated O2 photoreduction at photosystem I acceptor-side provides photoprotection to conifer thylakoids in early spring
Source: Nat Commun. 2023 Jun 3;14:3210. doi: 10.1038/s41467-023-38938-z (PMC10239515; doi:10.1038/s41467-023-38938-z)

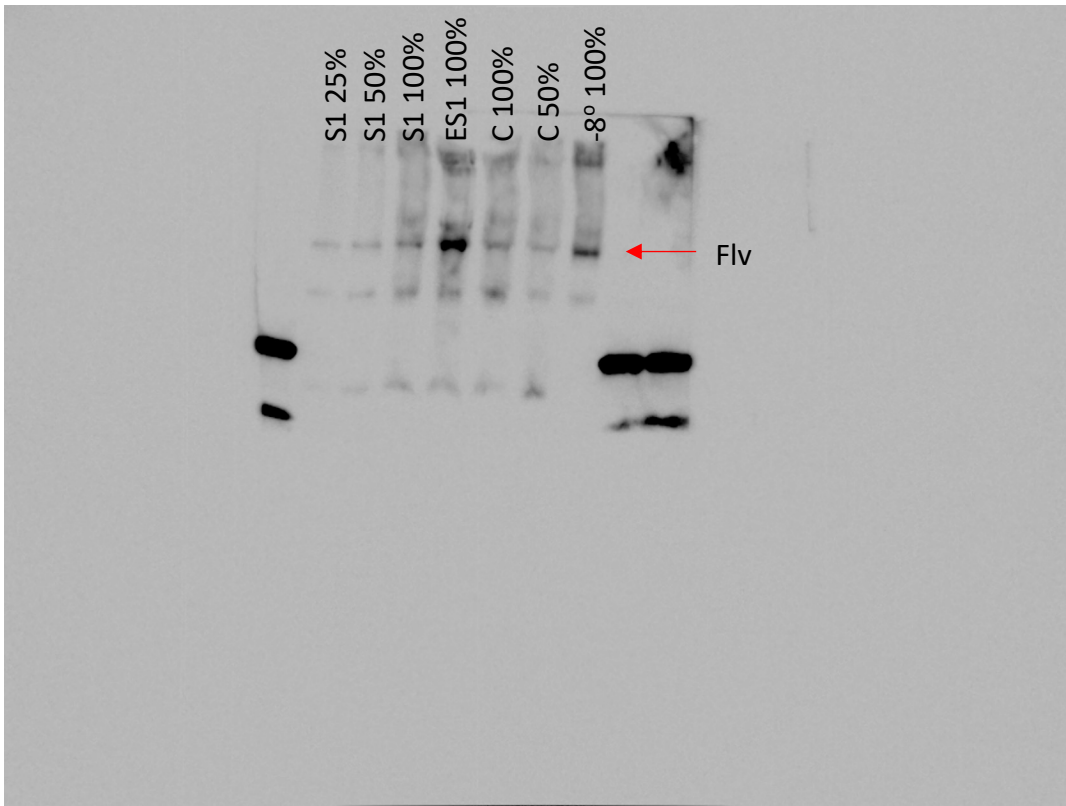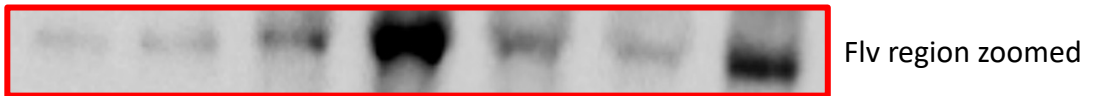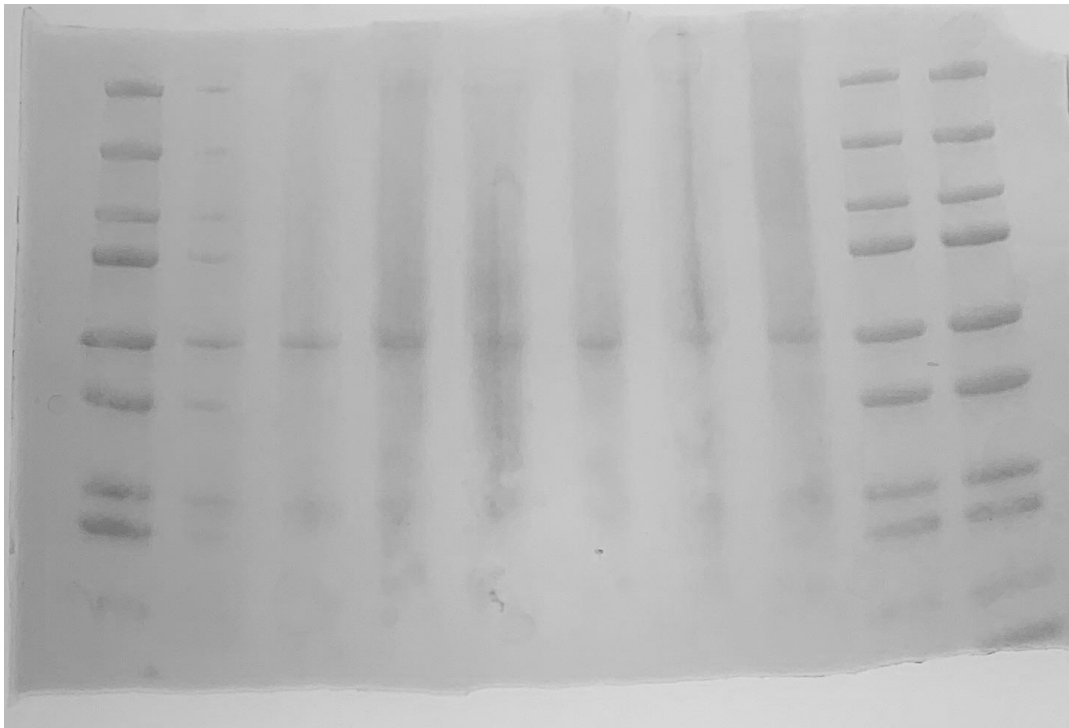

Gel scan in RGB

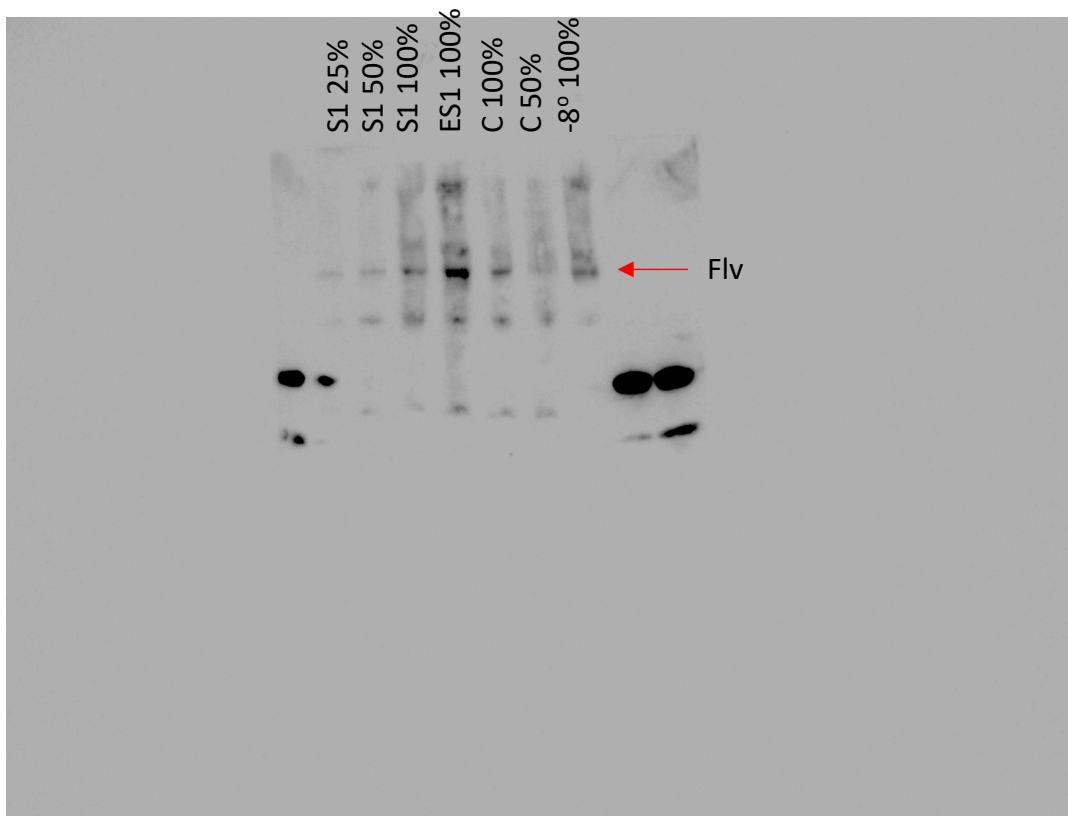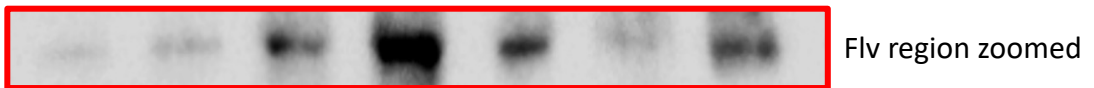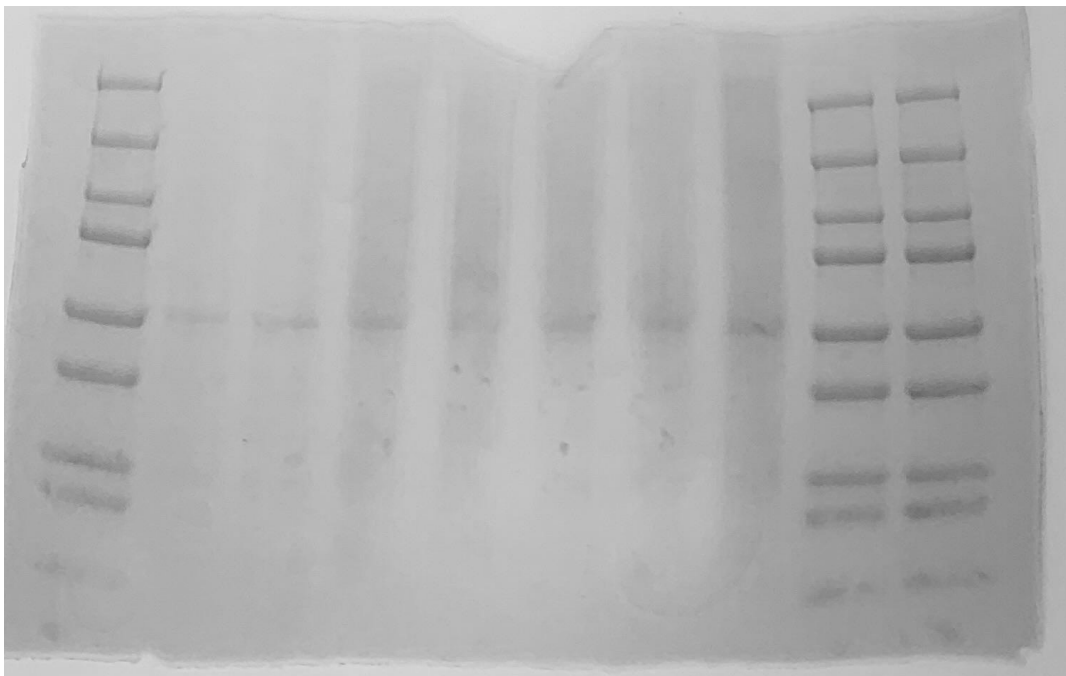

Gel scan in RGB

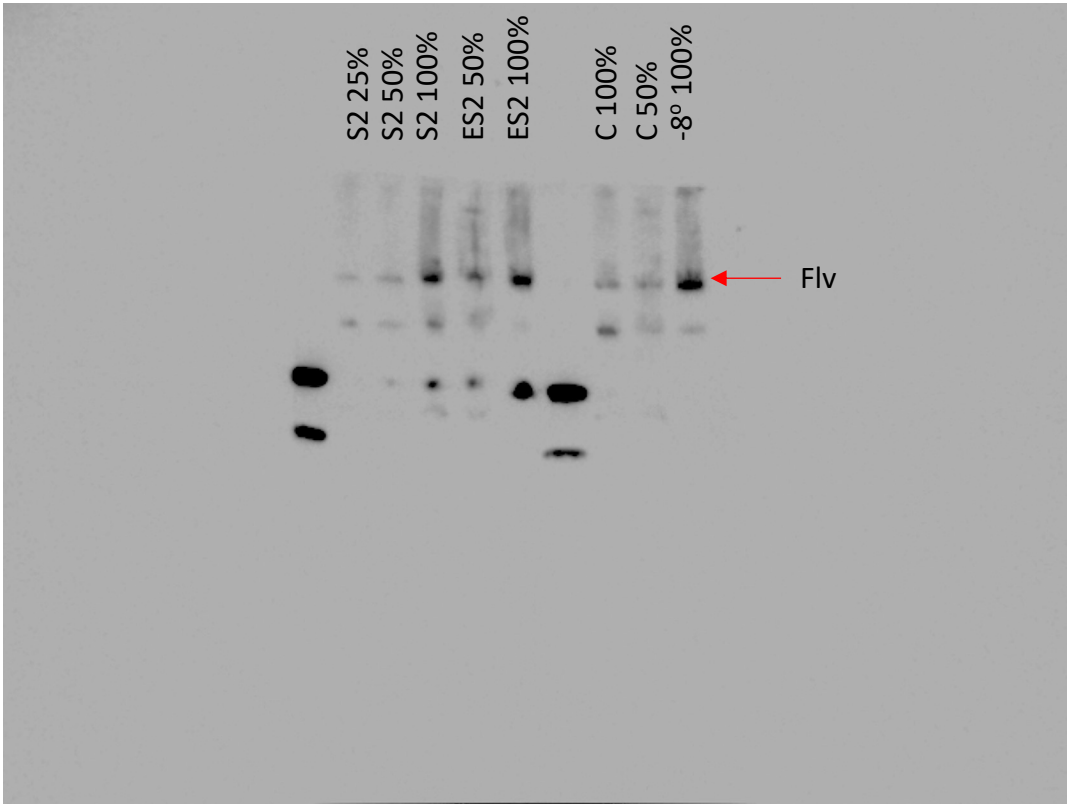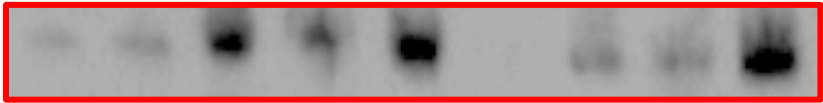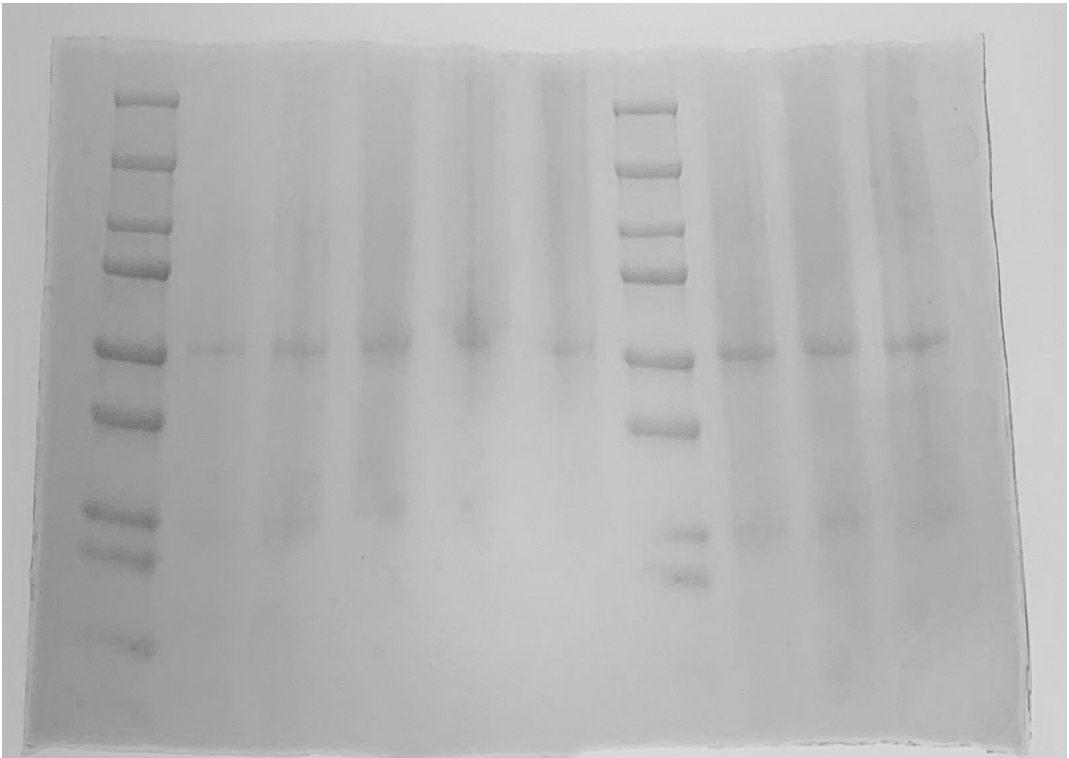

Gel scan in RGB

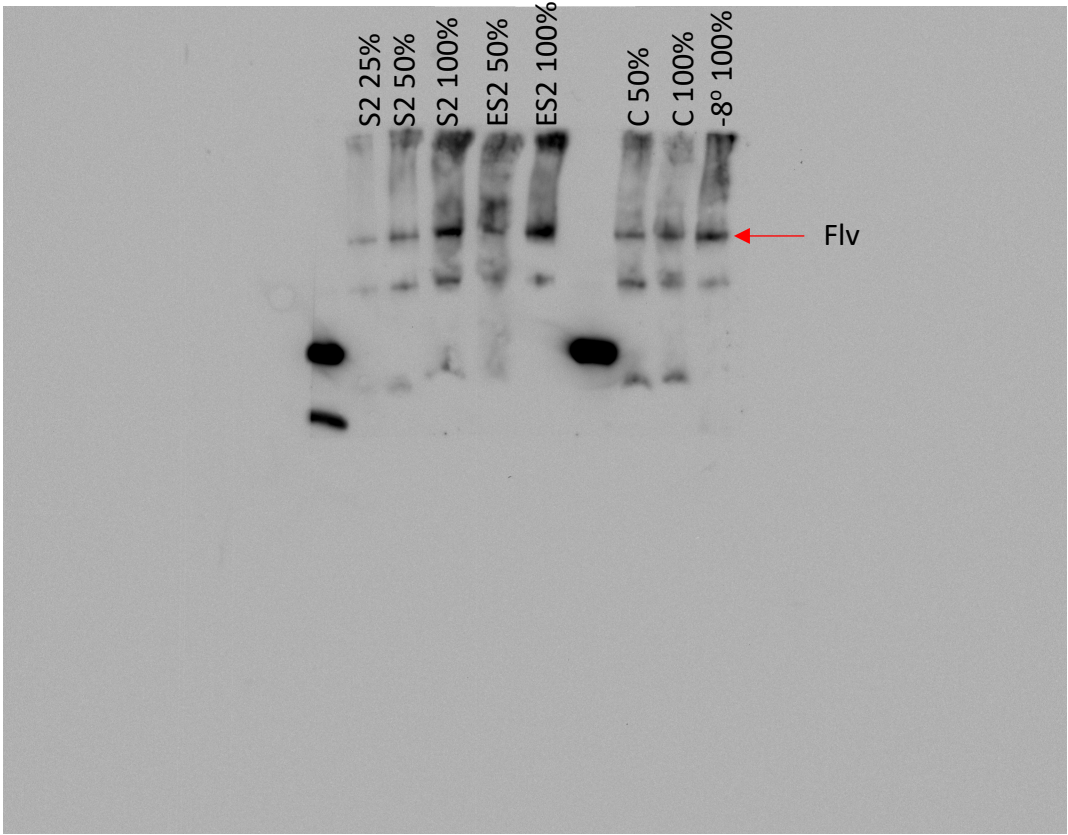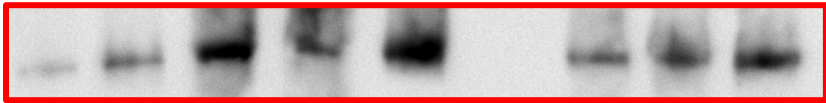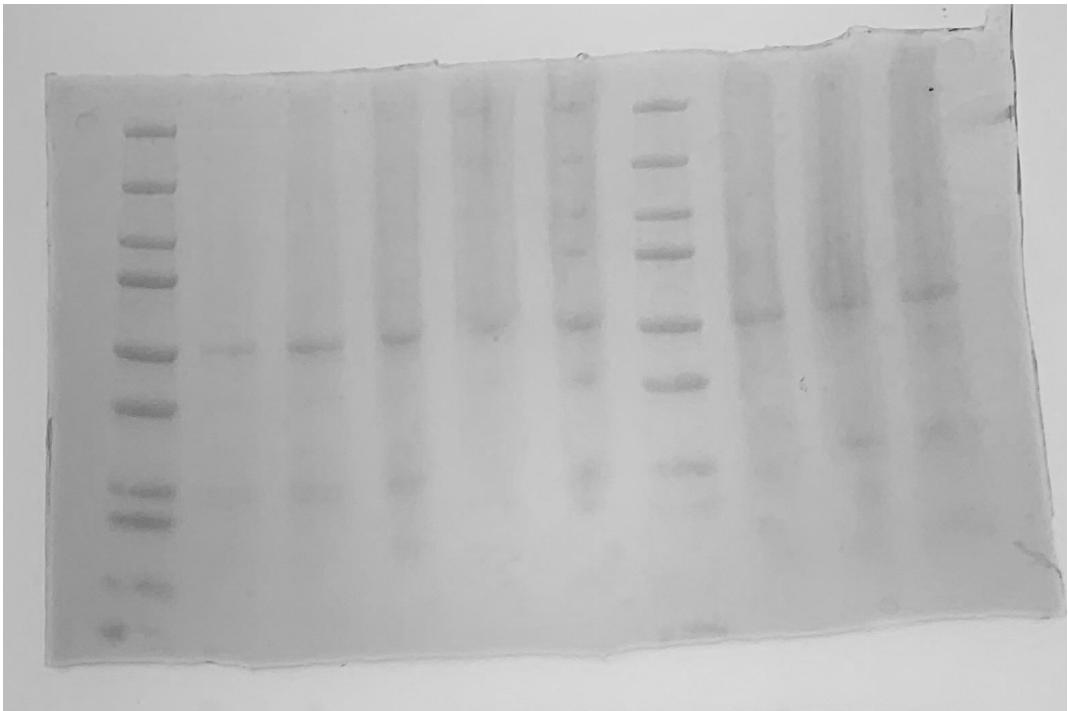

Gel scan in RGB

Supplement: Supplementary file 7 — Source Data [file 41467_2023_38938_MOESM7_ESM.zip › Raw data files/Supplementary figure 7/Flv blot replicates chamber.pdf]
